# Impact of microlearning on developing soft skills of university students across disciplines

**DOI:** 10.3389/fpsyg.2025.1491265

**Published:** 2025-04-25

**Authors:** Haozhun Luo, Weiyan Li

**Affiliations:** ^1^Faculty of Education, Southwest University, Chongqing, China; ^2^School of Music, Southwest University, Chongqing, China

**Keywords:** microlearning for various academic disciplines, soft skill development of university student, teamwork and leadership skills, communication and time management skills, emotional intelligence, China

## Abstract

**Introduction:**

This study explores the effectiveness of microlearning in developing key soft skills among university students across four academic disciplines: humanities and arts (HA), business studies (BS), medical sciences (MS), and technical and engineering (TE). Addressing the disconnect between academic training and industry expectations, the research investigates how microlearning interventions influence the development of teamwork skills (TWS), leadership skills (LS), communication skills (CS), time management skills (TMS), and emotional intelligence (EI). The study also aims to identify which disciplines benefit most from microlearning for each specific skill.

**Methods:**

A total of 384 Chinese university students participated in this study, with a questionnaire recovery rate of 93.23% and near-equal representation from each discipline. Participants completed pre- and post-intervention surveys following tailored microlearning modules. Statistical analyses—including paired sample *t*-tests, independent sample *t*-tests, and effect size calculations—were employed to test five hypotheses related to soft skill development across disciplines.

**Results:**

Findings indicate that leadership-focused microlearning modules significantly benefited TE and MS students, while EI training was particularly effective for BS students. Notable improvements in CS and TMS were observed among BS and TE students, aligning with skills demanded in corporate project management. Overall, microlearning interventions produced measurable enhancements in specific soft skills, with variation across academic disciplines.

**Discussion:**

The results suggest that integrating structured, discipline-specific microlearning into university curricula can effectively bridge academic-industry skill gaps. Faculty are encouraged to adopt scenario-based microlearning strategies to enhance student engagement. Higher education institutions should prioritize microlearning experience in student development and recruitment. Additionally, EdTech providers are urged to develop AI-powered interactive platforms to personalize learning, while students should proactively engage in targeted microlearning to improve academic and career outcomes.

## 1 Introduction

In today's rapidly evolving job market, soft skills are as crucial as technical expertise. Employers increasingly seek graduates, who possess the domain-specific knowledge along with interpersonal and cognitive abilities, such as teamwork skill (TWS), leadership skill (LS), communication skill (CS), time management skill (TMS), and emotional intelligence (EI) (Li et al., 2024). Many articles illustrate that these skills are essential for young adults to collaborate effectively, solve problems, and adapt to dynamic professional environments (Hamzah et al., 2024).

### 1.1 Research background

Despite the importance of soft skills in shaping the professional and personal success of young adults, traditional university education continues to fall short in providing structured training for these essential competencies. Universities predominantly emphasize technical expertise and theoretical knowledge, while often side-lining the development of soft skills (Romanenko et al., 2024). Thus, even though young adults are graduating with strong academic credentials, they need to face difficulties adapting to workplace environments that demand effective interpersonal skills, decision-making abilities, and collaboration. In an era where automation and artificial intelligence are reshaping industries, the ability to think critically, manage time efficiently, and lead teams has become vital as subject-specific expertise. Besides, employers increasingly prioritize candidates, who demonstrate well-rounded skill sets. However, many graduate young adults struggle to meet these expectations due to the limited focus on soft skills in their academic training (Park, 2024).

In this scenario, many universities attempt to bridge these gaps by providing skill-development workshops, mentorship programs, and/or extracurricular activities, whereas these initiatives often lack systematic integration within academic curricula. Without a structured and scalable approach, universities risk producing graduates, who excel in theoretical knowledge yet struggle with real-life oriented soft skills (Gruber et al., 2022). Addressing this concern requires a shift toward embedding structured soft skills training into university education, with microlearning emerging as a useful solution to enhance skill acquisition in a more engaging, accessible, and measurable manner. Furthermore, collaboration with industry professionals and employers can provide valuable insights into the specific soft skills required in the workforce (Romero-Rodríguez et al., 2023).

### 1.2 Impact of microlearning to enhance soft skills

In recent years, microlearning, characterized by short, focused, and interactive learning modules, has emerged as a promising approach to skill development. Unlike conventional lecture-based instruction, which typically provides information in lengthy sessions that lead to cognitive overload, microlearning segments the learning into bite-sized units that are easier to process and retain (Silva et al., 2025). This method aligns with cognitive learning theories, such as spaced repetition, which enhances long-term retention by exposing learners to information at strategic intervals; cognitive load theory, which suggests that breaking complex information into manageable parts reduces mental fatigue and increases understanding; and experiential learning, which emphasizes learning through direct experience and reflection (Corbeil et al., 2023). These principles collectively support the notion that microlearning through short bursts facilitate improved knowledge retention and skill acquisition for young adults.

Nevertheless, this study finds that while microlearning has been widely adopted in corporate training and technical education, whereas its potential for developing specific soft skills in university students has barely been explored. Many established articles predominantly focus on the impact of microlearning on knowledge acquisition, such as language proficiency, IT certifications, and compliance training, rather than the development of various interpersonal and professional competencies (Hlazunova et al., 2024). Unlike purely theoretical subjects, soft skills require continuous practice, real-world application, and self-reflection, all of which microlearning potentially supports through interactive simulations and scenario-based learning. However, there is a lack of comprehensive studies on whether microlearning cultivate the soft skills, particularly TWS, LS, CS, TMS, and EI, effectively within higher education contexts (Alias and Razak, 2024). Given the growing importance of soft skills in career success, there is a need to evaluate the impact of microlearning on university students, specifically from diverse academic disciplines. Also, this study plans to determine whether discipline-specific customization of microlearning strategies is necessary for improving soft skills of university students.

### 1.3 Effectiveness of microlearning across academic disciplines

A number of articles describe that the effectiveness of microlearning in developing soft skills among university students is hardly uniform across academic disciplines. Each field of study follows distinct cognitive approaches, learning preferences, and professional competencies, thereby making a standardized microlearning framework inadequate. By tailoring microlearning strategies to align with discipline-specific expectations, students can cultivate essential soft skills in ways that are most relevant to their academic training and future careers (Xu et al., 2024). Focusing on four key disciplines, namely humanities and arts (HA), business studies (BS), medical sciences (MS), and technical and engineering (TE), this study plans to examine how microlearning can be customized to enhance aforementioned five critical soft skills of young adults.

For instance, in the HA discipline, researchers find that creativity, critical interpretation, and interpersonal engagement are fundamental to both academic success and professional growth (Romanenko et al., 2024). They describe CS and EI to be particularly crucial for HA students, because their work often involves abstract concepts, subjective analysis, and complex human interactions. Notably, effective storytelling, argumentation, and persuasion rely on strong communication abilities, while emotional intelligence enhances sensitivity to diverse perspectives and collaborative work (Gao et al., 2024). Microlearning modules in HA can thus incorporate discussion-based activities, interactive storytelling, and role-playing exercises that strengthen these skills by simulating real-world creative and social interactions. In contrast, BS students require CS and EI to navigate corporate environments, manage relationships, and persuade stakeholders. Strategic decision-making in business scenarios are closely tied to an individual's ability to communicate effectively and interpret emotional cues in negotiations. Microlearning can support BS students through scenario-based training, gamified simulations of business negotiations, and short case studies that reinforce the practical application of interpersonal and strategic thinking skills in business contexts (Adeoye et al., 2024).

On the other hand, many articles describe that LS and TMS play a pivotal role for students in MS group in preparing them for leadership roles in healthcare management, medical administration, and organizational structures. Effective leadership is essential for managing healthcare teams, thus making critical decisions under pressure, and ensuring ethical and patient-centered care (Shamir-Inbal and Blau, 2022). Also, TMS can have a crucial role to balance patient care, research, and administrative responsibilities for medical students. Therefore, there is a scope to investigate whether microlearning strategies for MS students should include quick decision-making exercises and interactive time management workshops be tailored to real-world medical and healthcare scenarios. Likewise, this study seeks to find the priority soft skill of TE students to manage large-scale technical projects, lead engineering teams, and meet deadlines in high-pressure environments. Also, one should determine if microlearning interventions for TE students shall incorporate task-based learning and project management simulations.

### 1.4 Research questionnaire

This study explores the impact of microlearning on various soft skills across different academic disciplines, thereby aiming to assess whether a tailored microlearning approach shall effectively enhance skill development and addressing the unique challenges of university students in diverse academic and professional contexts. This study seeks answers to the following research questions:

- How does microlearning influence the development of specific soft skills, like TWS, LS, CS, TMS, and EI, among university students?
  With its answer, this study shall address the general impact of microlearning and examine whether this tool significantly enhances major soft skills of university students.
- To what extent do students from different academic disciplines, namely HA, BS, MS, and TE, experience varying improvements in soft skills through microlearning interventions?
  This question explores the effectiveness of microlearning differing across academic disciplines, while exploring whether soft skill development is uniform on the field of study.
- Which academic disciplines experience the significant improvements in LS and TMS due to the microlearning intervention?
  Given the structured and goal-oriented nature of these disciplines, answer to this question examines whether microlearning enhances LS and TMS more effectively for MS and TE groups of students than the rest.
- Also, which academic disciplines experience considerable gains in TWS and EI post-intervention of microlearning?
  This question investigates whether BS and HA students benefit properly in developing TWS and EI through microlearning, considering that their disciplines emphasize interpersonal engagement, negotiation, and emotional awareness.
- Does microlearning lead to notable improvements in CS and TMS among students of BS and TE disciplines?
  As BS students rely on effective CS for business interactions and TE students require efficient TMS for technical problem-solving, this question assesses whether microlearning effectively enhances CS and TMS in these two groups.

By addressing above questions, this study shall tackle the critical issue of evaluating the effectiveness of microlearning across different academic disciplines and assessing the potential need for its customization.

### 1.5 Organization of the paper

The remainder of this paper is structured as follows: Section 2 provides the review of recent and established literature across four key aspects and briefly describes research gaps. Next, Section 3 designs five hypotheses that address the research gaps identified in this study. The research methodology, including participants' details and research instruments, is outlined in Section 4. Then, Section 5 reports the results of various statistical tests, while Section 6 evaluates the validity of the proposed hypotheses. This study extracts several actionable insights for relevant stakeholders and discusses their global implications therein. Also, this study compares the impact of microlearning with traditional and experiential learning approaches. Finally, Section 7 summarizes the key findings, acknowledges its limitations, and proposes directions for future research.

## 2 Literature review

This section provides a brief review of established literature on four major aspects, while subsequently discussing the major research gaps as follows:

### 2.1 Microlearning impacting communication and time management skills

Analysts describe that microlearning improves CS and TMS by delivering concise, targeted lessons that enhanced information retention and practical application (Alias and Razak, 2024). Thus, young adults in colleges and universities should develop clearer communication strategies and better organizational habits, which would enable them to manage tasks efficiently and adapt to dynamic professional and academic environments (Taylor and Hung, 2022).

Among recent articles, Fitria (2022) examined the applications, benefits, and limitations of microlearning through library research. Their analysis revealed that microlearning utilized videos, applications, gamification, infographics, and social media for content delivery, whereas they established that microlearning would enhance retention, comprehension, and flexibility by offering short, customizable lessons. However, their investigation found its unsuitability for complex topics and the challenge of content creation for educators. Also, they emphasized that effective implementation depended on learner characteristics, teacher readiness, and resource availability. Javorcik et al. (2023) examined strategies in a microlearning course for teachers at the University of Ostrava. Tracking 378 students through learning analytics, they identified six approaches to successful course completion, while being unaffected by gender yet varying in completion time. They performed the cluster analysis and heatmaps to reveal students' behavior, learning material, and device usage. Also, they emphasized the need for personalized learning strategies to enhance microlearning effectiveness in teacher education.

Around this time, Nikkhoo et al. (2023) examined the foundation of microlearning in Ebbinghaus' forgetting curve, while demonstrating its effectiveness in improving knowledge retention by delivering content in small, spaced segments. In that article, various digital formats, including videos and podcasts, facilitated just-in-time learning across devices, even while they found microlearning to be unsuitable for complex topics requiring deep analysis. Also, Fitria (2022) examined the applications, benefits, and limitations of microlearning through library research. Their analysis revealed that microlearning utilized videos, applications, gamification, infographics, and social media for content delivery, whereas they established that microlearning would enhance retention, comprehension, and flexibility by offering short, customizable lessons. However, their investigation found its unsuitability for complex topics and the challenge of content creation for educators. Although the term microlearning emerged in 2005, Corbeil et al. (2023) described that its use and research resurged as adult learners became more mobile and juggled competing priorities. They examined the effectiveness of micro-lessons on university students' knowledge acquisition and skill performance.

Very recently, Gasca-Hurtado et al. (2024) designed and implemented a microlearning strategy that was supported by a mobile application. Their approach was useful to enhance motivation and learning outcomes in software project management using the Scrum framework. They conducted a quasi-experimental study with pretest–post-test measurements to assess its effectiveness, whereas they could identify that participants would show significantly higher motivation after using the application. Thus, they advocated for the potential of said mobile application in supporting microlearning. In this line, Monib et al. (2024) examined the impact of microlearning on learning outcomes by analyzing quantitative and qualitative data from lifelong learners. They identified content and learner-level factors influencing outcomes, categorized into contextual, behavioral, cognitive, and affective domains. Using SmartPLS and ATLAS.ti, they could reveal that media richness should enhance interactivity, engagement, satisfaction, and comprehension, whereas the challenges included limited peer interaction and comprehension difficulties.

Moreover, Rahbar et al. (2024) investigated the impact of social media-based microlearning on knowledge, self-care, and self-efficacy among patients with type 2 diabetes in an Iranian hospital. They assigned 80 patients to either social media-based microlearning or conventional training group. Their post-intervention analysis showed significantly higher improvements in the social media-based microlearning group (*p* < 0.001). Thus, they established microlearning as an effectively tool to enhance self-efficacy, self-care, and knowledge in diabetes management. In a software engineering study, Gasca-Hurtado et al. (2024) designed and implemented a microlearning strategy using a mobile application to evaluate motivation and learning outcomes in software project management with Scrum. Their quasi-experimental pretest–post-test study found significantly increased motivation yet minimal statistically significant improvement in learning outcomes. Their findings showed the potential of microlearning to enhance motivation while supporting learning in software engineering education. Around this time, Kohnke et al. (2024) examined microlearning in online and blended formats for teacher professional development in Hong Kong. They involved 32 pre-service English teachers, using questionnaires, interviews, and observations. They showed that participants faced challenges in technology integration, task design, and time management. Thus, they recommended personalized, hands-on training while emphasizing the need for conceptual frameworks to support microlearning.

### 2.2 Cognitive load theory and impact of microlearning on teamwork skill

The bite-sized learning approach, microlearning, aligns with the well-established cognitive load theory by reducing extraneous cognitive load and enhancing retention. With concise and focused content, researchers find that microlearning optimizes working memory, prevents overload, and improves learning efficiency, thus making this effective for skill acquisition and knowledge retention (Alias and Razak, 2024).

In recent years, Khong and Kabilan (2020) developed a theoretical model for microlearning in second language (L2) instruction to address the lack of theoretical grounding in ML adoption. They analyzed the benefits and limitations of machine learning, examined three established theories, and integrated them into a conceptual framework. They clarified the application of machine learning in L2 learning, which showed its pedagogical potential. Gerbaudo et al. (2021) developed an online video model for IT professionals' continuing education using the design thinking methodology. They conducted a five-stage process, namely empathy, definition, ideation, prototype, and testing, to align video content with user needs, whereas they tested a prototype with 150 participants through a self-assessment survey. Around this time, Susilana et al. (2022) investigated the application of microlearning strategies in online learning to mediate students' cognitive load. Using a qualitative approach with 45 student volunteers, they collected data through questionnaires, interviews, and document analysis. Those findings revealed that microlearning effectively reduced intrinsic and extraneous cognitive load while enhancing germane cognitive load. Their study emphasized the impact of microlearning in optimizing online curriculum development, while leading to improved student learning outcomes.

Around this time, Sozmen (2022) analyzed the benefits and limitations of microlearning as a digital teaching technique. They indicated that breaking content into smaller segments enhanced student engagement and facilitated learning, whereas microlearning supported on-demand learning and daily integration through diverse activities. However, they noted that its success depended on learners' characteristics, teachers' digital proficiency, and external factors like material accessibility, while advocating for the subject-specific microlearning method selection. Javorcik et al. (2023) investigated students' approaches in a microlearning course at the University of Ostrava's Faculty of Education. They conducted the research in 2021/2022 by analyzing research data from 378 students using learning analytics and cluster analysis. Their results identified six distinct study strategies, which could remain unaffected by gender yet differing in completion time. They showed the potential of personalized microlearning to enhance teacher education effectiveness. Also, Shamir-Inbal and Blau (2022) examined self-regulated learning processes, strategies, and challenges in a microlearning-based blended course for 172 Israeli ICT leaders. Their data analysis confirmed the usefulness of cognitive and emotional perceived learning. They conducted the multiple regression analysis and thus explained 16.2% of teachers' professional development achievement variance and 48.4% of willingness for future microlearning-based professional development. They established the utility of self-regulated learning theory, perceived learning frameworks, and teachers' professional development practices.

In this regard, Hlazunova et al. (2024) examined video-based microlearning in teaching information systems and technologies in economics at the National University of Life and Environmental Sciences of Ukraine. Whereas they assessed e-course quality and student performance, their results from 39 respondents showed 75% perceived microlearning as effective. Since they found some improved learning outcomes and positive evaluations from both students and teachers, they promoted the role of video-based microlearning in enhancing educational quality. Alias and Razak (2024) examined microlearning strategies and their impact on learning in the digital age through a systematic literature review. They analyzed various microlearning techniques, which included bite-sized content, mobile learning, video-based learning, and social learning, while assessing their effectiveness across contexts. They identified challenges in implementing microlearning and proposed solutions, and showed benefits, and strategic recommendations for educators and organizations seeking to enhance learning outcomes through microlearning based digital education strategies. Takhdat et al. (2024) investigated the effects of a brief mindfulness meditation training program on anxiety and cognitive load in emergency simulation training among health professions students. Seventy participants were randomly assigned to an experimental or control group, in which the experimental group exhibited lower state-anxiety and reduced cognitive load with improved TWS in simulation assessments. Nevertheless, they found hardly any significant differences in trait-anxiety and mindfulness were observed at the six-month follow-up. Moreover, Hung et al. (2024) compared the effectiveness of synchronous and asynchronous online teaching in dermatology lectures for undergraduate medical students. Among 170 participants of their study, 70 participants chose synchronous (i.e., live Webex lecture) and 100 participants chose asynchronous (i.e., YouTube videos). They found that both methods improved post-test and retention scores, with no significant difference in satisfaction levels.

In a novel research, Wang et al. (2024) investigated the impact of feedback in a VR learning environment on cognitive load and hands-on task performance in STEM education. Their quasi-experiment assigned participants to VR learning with or without feedback, whereas their results showed that feedback reduced extraneous cognitive load, increased engagement, and improved performance in physical hands-on tasks. In that article, participants with feedback required fewer trial-and-error attempts, thus demonstrating the effectiveness of structured guidance in VR-based learning. Silva et al. (2025) analyzed microlearning in basic education, identifying commonly used digital technologies, learning theories, and social technologies, whereas they searched multiple databases, selected studies based on eligibility criteria, and conducted qualitative analysis. They showed that microlearning, integrated with digital tools, improved motivation, performance, and engagement. Around this time, Li et al. (2024) examined the effects of human-human and human-machine collaborative learning on student teachers' STEM teaching performance. They had continued a span of two months, in which 23 student teachers participated in weekly 3-h sessions. Their results showed that those using ChatGPT demonstrated higher critical thinking, task efficiency, and lower cognitive load, while those paired with in-service teachers performed slightly better in final teaching design proposals. This way, they advocated for the potential of AI in collaborative learning and professional development.

### 2.3 Microlearning impacting leadership skill and emotional intelligence

Microlearning enhances LS and EI in young adults by developing self-awareness and decision-making (Murry et al., 2024). The bite-sized learning modules in microlearning enhance CS and TWS, while guiding young adults to develop emotional resilience and leadership capabilities, and preparing them for dynamic social environments (Pham et al., 2024).

In this regard, Arnab et al. (2021) examined the use of microlearning to address cultural risks in multicultural workplaces through an online platform featuring 15 mini-games. Based on a survey of 154 employees across five countries, they identified eight key risk areas. Next, by testing with 166 participants, they could confirm the effectiveness of the platform in developing cultural awareness and self-assessment. Their results contributed to pedagogical and gameful design considerations for developing engaging micro-learning resources. Garshasbi et al. (2021) explored the integration of microlearning and computer-supported collaborative learning to enhance interaction and retention in online learning. They identified challenges in learner engagement, content interaction, and collaboration, whereas they showed the potential of intelligent learning environments to replicate classroom conditions. By synthesizing ML and CSCL principles, they provided STEM educators with a roadmap for developing comprehensive and effective online learning platforms. Shamir-Inbal and Blau (2022) explored self-regulated learning processes, strategies, and challenges in microlearning within blended courses aimed at enhancing pedagogical-technological knowledge among 172 Israeli ICT school leaders. Their regression analysis revealed that teaching and training seniority, along with cognitive perceived learning, influenced TPD achievement. Also, they found that perceived learning was a predictor of willingness for future participation. Around this time, Romero-Rodríguez et al. (2023) examined students' creative competence before and after a microlearning experience involving 57 Spanish and Mexican university students. Using a quasi-experimental design with pre- and post-tests, they found that COIL activities significantly enhanced creative competence.

Of late, Abbasalizadeh et al. (2024) assessed the effectiveness of a microlearning-based mHealth application in reducing stress and anxiety among ICU nurses through a randomized controlled trial with 60 nurses in Tehran. They found that the intervention group, which received resilience training via the application, experienced significant reductions in stress and anxiety, whereas their control group showed increases. This way, their results could support the application's effectiveness and recommended its use as an innovative educational tool for improving ICU nurses' wellbeing. Adeoye et al. (2024) found that within the context of higher education, particularly in microlearning and micro-credentials, mindfulness, compassion, and Ubuntu would play a pivotal role in driving a transformative shift. Thus, mindfulness practices should develop engaged awareness, compassion enhanced empathy, and Ubuntu strengthened social connectivity. Also, they described that their integration into curricula and pedagogy promoted social responsibility and leadership, while facilitating meaningful educational and personal development. Also, Xu et al. (2024) described that children on the spectrum often required both formal services and unpaid caregiver support. Like, a U.S. autism surveillance study reported an increase in racially/ethnically diverse autistic children, who, alongside their caregivers, faced barriers in accessing services. Thus, they determined that structural challenges led to caregiver stress, while impacting service management. They suggested to use microlearning to adapt *Parents Taking Action*, which had been a culturally responsive intervention, into bite-sized modules to support overwhelmed caregivers more effectively.

### 2.4 Role of microlearning on various academic disciplines

In recent years, academic institutions are increasingly and effectively implementing microlearning across various academic disciplines, including engineering, healthcare, humanities, and business, for enhancing engagement and retention of young adults through concise, targeted content (Denojean-Mairet et al., 2024). This approach supports skill development and knowledge acquisition to diverse learning needs and professional demands (Gruber et al., 2022).

Among established articles, Skalka et al. (2021) explored a conceptual framework by integrating microlearning and automatic code evaluation to enhance programming education. Their framework provided immediate feedback and engaged students in developing virtual learning environments, whereas their quantitative study assessed its effectiveness. Notably, they found hardly any improvements in first semester students yet determined notable progress in advanced courses as the effects of microlearning. Thus, they advocated for the potential to support programming education and sustain modern software development practices. Mozgalova et al. (2021) analyzed the significance of soft skills for future specialists in music and the arts, while emphasizing their distinction from hard skills. They examined theoretical materials to identify essential interpersonal competencies for music educators. Their findings advocated teachers to integrate soft skills into training, despite challenges in balancing them with technical instruction. This way, they supported the importance of soft skills in preparing students for professional success. Also, Joia and Lorenzo (2021) examined the factors influencing the effectiveness of technology-mediated courses during the COVID-19 pandemic. They found that teachers' digital competence and meta-cognitive support significantly impacted pedagogical success. Also, they revealed that hard skill disciplines faced greater challenges in achieving learning objectives when transitioning to digital environments compared to soft skill disciplines.

Very recently, Romanenko et al. (2024) investigated the alignment between soft skills training and employer expectations at ITMO University without considering any particular branch of education. They analyzed job vacancies, surveyed students and employers, and examined labor market trends in Russia's Northwestern Federal District, whereas they established that employers should value soft skills and integrate those into the curriculum to boost students' career prospects. Also, they emphasized the importance of continuing soft skills development to enhance graduates' employability and workplace effectiveness. Hamzah et al. (2024) investigated the soft skills transfer problem and developed the COMPASS model, while integrating Baldwin and Ford's training transfer framework with behavior change model. Mapping various factors onto the COMPASS model, they confirmed its applicability. Also, they demonstrated that model components would contribute to training transfer and improve soft skills application in professional settings for any discipline. Also, Misra et al. (2024) examined job opportunities and career empowerment in emerging markets for any graduate adults, emphasizing the role of technology and industry collaboration in training programs. They analyzed case studies of effective models, thus showing their impact on workforce readiness and economic growth. They established the importance of aligning training with employment trends and promoting continuous professional development, whereas they could design impactful programs that supported economic development and career advancement for young adults irrespective of their discipline. Veeramanickam et al. (2025) investigated the impact of digital media and smart campus infrastructure on e-learning during and after COVID-19. They examined infrastructural challenges and platform utilization for enhanced accessibility, whereas they found increased online course enrolment and improved success rates. Also, they found that gamification and flipped classrooms significantly enhanced learner engagement, and proved the importance of gamified learning applications in improving satisfaction and learning competence of any graduate young adult.

### 2.5 Research gaps

Despite the growing body of literature on microlearning, current review identifies a significant research gap concerning its impact on developing various soft skills across different academic disciplines. Established articles discussed the effectiveness of microlearning in enhancing LS and EI by developing self-awareness and adaptability among learners (Murry et al., 2024; Pham et al., 2024). However, those articles rarely examined whether improvements in these skills varied across disciplines, namely HA, BS, MS, and TE. Given that soft skills are context-dependent and shaped by discipline-specific cognitive processes, this study notes that a broad, undifferentiated approach to microlearning shall often fail to capture the unique developmental trajectories of students in different fields. Thus, addressing this gap is essential, because discipline-specific soft skill training can better prepare students in professional and personal landscapes.

Moreover, there is a limited body of statistical and survey-based evidences on whether microlearning consistently enhances LS and TMS in the highly structured disciplines of MS and TE. While existing articles demonstrated that microlearning had improved cultural awareness (Arnab et al., 2021), and self-regulated learning (Shamir-Inbal and Blau, 2022), its role in developing LS and TMS within MS and TE programs was hardly explored. Moreover, since MS students are often required to manage teams and make strategic decisions, while TE students must efficiently coordinate projects and meet technical deadlines, this study advocates for the need for a deeper understanding of how microlearning influences these skills within those academic disciplines to inform more effective curriculum design.

Likewise, this study finds that although numerous articles considered that microlearning would positively impact CS and TMS (Taylor and Hung, 2022; Alias and Razak, 2024), a little attention has been paid to its differential effects across academic disciplines. Current research indicates that microlearning develops personalized learning experiences (Javorcik et al., 2023) and increases motivation (Gasca-Hurtado et al., 2024). However, there are scopes to investigate whether these advantages would translate into distinct skill development patterns across disciplines. Like, BS and TE students typically rely heavily on CS and TMS for professional success, yet existing studies fail to clarify whether microlearning interventions yield significant improvements in these areas for these student groups. Likewise, while EI is a crucial competency for BS and HA students, who frequently engage in interpersonal communication and negotiation (Mozgalova et al., 2021), there is a lack of research investigating whether these students experience the highest gains in EI through microlearning interventions.

In addition, the alignment of microlearning with cognitive load theory indicated that this had the potential to optimize learning efficiency (Alias and Razak, 2024). Some researchers have explored its role in reducing cognitive overload (Sozmen, 2022; Hlazunova et al., 2024), yet very few articles can be found to examine whether its effectiveness varies significantly across disciplines. Given that students in different fields process and apply knowledge in distinct ways, microlearning should support skill acquisition at different rates across disciplines (Gruber et al., 2022). However, there is insufficient empirical evidence on whether HA, BS, MS, and TE students develop soft skill competencies through microlearning in unique ways, thus showing the need for further research in this area.

By addressing these gaps, this study aims to provide a deeper understanding of the role of microlearning in soft skill development. Specifically, this study investigates whether MS and TE students experience the most significant improvements in LS and TMS, whether BS and HA students exhibit the highest gains in EI, and whether BS and TE students show notable progress in CS and TMS. This way, current research advances the theoretical understanding of microlearning in higher education while showing practical strategies for designing discipline-specific microlearning that meets the professional and cognitive demands of various academic fields.

## 3 Framework of the proposed hypotheses

Building on the aforementioned discussion on microlearning influencing soft skills, this study proposes five hypotheses as follows.

### 3.1 Role of microlearning to enhance soft skills of university students

In recent years, microlearning has emerged as a widely recognized and effective pedagogical approach. By delivering content in concise, targeted segments, this tool promotes active participation, encourages self-directed learning, and facilitates the application of soft skills among young adults. In this context, soft skills refer to the personal attributes that are essential for overall success and personal growth. Specifically, this study examines how microlearning enhances five key varieties of soft skills, namely LS, CS, TWS, TMS, and EI. These skills are essential for university students as they navigate academics, transition to professional life, and thrive in diverse environments (Hamzah et al., 2024).

Researchers identify that microlearning effectively develops LS by promoting real-world challenges in concise, engaging formats (Shamir-Inbal and Blau, 2022). In corporate sector, training programs, like Google's Whisper Courses, integrate short leadership lessons into employees' daily routines. This aspect promotes that university students can earn benefits through microlearning-based leadership modules featuring case studies, decision-making exercises, and interactive simulations. These activities shall equip them with the ability to make quick yet informed decisions, delegate tasks efficiently, and build confidence in high-pressure situations (Mozgalova et al., 2021). Meanwhile, CS have become increasingly vital in today's digital and globalized world. Microlearning enhances these skills through interactive video lessons, role-playing exercises, and real-time feedback mechanisms. Just as Duolingo's microlearning model helps users master new languages through short, gamified lessons, this study finds that microlearning-based courses on web-based platforms significantly improve young adults' ability to articulate ideas concisely, actively listen, and engage in meaningful conversations. As a result, they report increased confidence in both professional and academic communication (Romanenko et al., 2024).

For decades, analysts have emphasized that TWS and TMS are essential for success for young adults. Microlearning enhances TMS by providing flexible, structured learning that fits seamlessly into university students' daily routines, whereas web-based learners can improve task breakdown, time allocation, and continuous learning habits (Takhdat et al., 2024). Pomodoro-based techniques, which emphasize focused, time-boxed learning, mirror microlearning's ability to reinforce time management. Companies like IBM incorporate microlearning into employee training to boost efficiency. Therefore, by adopting microlearning-driven time management strategies, young adults can better prioritize tasks, meet deadlines, and optimize study routines (Nikkhoo et al., 2023). Additionally, various studies deliberate on the crucial role of emotional intelligence in academic performance and career growth. Researchers find that microlearning develops emotional intelligence through scenario-based learning and real-world emotional regulation techniques. Whereas companies, like Johnson & Johnson, use microlearning to train employees in emotional intelligence by simulating workplace scenarios, universities can integrate short mindfulness and empathy-building exercises into learning systems with interactive stories helping students to develop empathy and emotional resilience (Murry et al., 2024).

This way, the present study finds that microlearning effectively develops critical soft skills for young adults across educational and professional contexts. Whether through corporate leadership programs, language-learning apps, productivity strategies, and/or emotional intelligence training, microlearning consistently proves its value in shaping well-rounded individuals (Garshasbi et al., 2021). Therefore, by integrating microlearning into academic curricula, universities can better prepare students for the demands of both professional careers and personal growth. Given these aspects, this study explores whether adapting microlearning to diverse educational settings can enhance university students' soft skills in general, thereby leading to the formulation of the following hypothesis:

*H*_1_: Microlearning enhances the development of soft skills among university students, including teamwork, leadership, communication, time management, and emotional intelligence.

### 3.2 Microlearning impacting leadership and time management across student groups

Universities worldwide are increasingly adopting microlearning to boost soft skills development among young adults. By delivering structured, targeted lessons in short bursts, microlearning enhances critical thinking, decision-making, and productivity, even while the differences in learning structures, cognitive demands, and professional expectations cause its effectiveness to vary across academic disciplines (Sozmen, 2022). A number of articles specifically describe that leadership development is influenced by students' academic and professional environments (Gao et al., 2024). For instance, students graduating in technical, engineering, and/or medical stream often work in high-pressure decision-making scenarios where leadership is a critical skill. TE students frequently oversee engineering projects, troubleshoot technical challenges, and collaborate within teams, thus requiring quick and informed decision-making. Microlearning supports their leadership development through scenario-based simulations that replicate real-world engineering challenges and thus strengthen their decision-making abilities. Likewise, MS students, particularly those in clinical or research settings, need strong leadership skills to manage patient care, coordinate medical teams, and make critical health-related decisions. For them, microlearning can provide diagnostic simulations and emergency response training tailored to the structured leadership demands of the medical field (Kouzes and Posner, 2012).

In contrast, whereas BS and HA students benefit from microlearning, the impact of microlearning on LS is likely less immediate for them. Business students are often exposed to leadership concepts through structured management theories and experiential learning, which makes their leadership development a gradual process rather than an immediate necessity. Microlearning reinforces LS through case studies and strategic decision-making exercises, even though this tool hardly replaces the experiential leadership growth that business students gain over time. Also, HA students are engaged in leadership through critical analysis, debate, and discourse rather than hands-on decision-making. While microlearning can support development of LS through interactive discussions and scenario-based learning, this study anticipates that its influence is likely lower since leadership in humanities is cultivated through long-term analytical and theoretical engagement rather than immediate problem-solving (Shamir-Inbal and Blau, 2022).

On the other hand, this study finds that the impact of microlearning on TMS should vary across student groups based on workload intensity, deadline constraints, and academic structures (Heath and Shine, 2021). TE and MS students face particularly demanding schedules, which require strict time management to balance coursework, research, and practical applications. TE students usually work on complex, deadline-driven projects, which make microlearning an effective tool for improving time efficiency through structured techniques, like Pomodoro-based study sessions, workflow optimization, and task segmentation. Again, MS students manage multiple responsibilities, from coursework and research to clinical work, where effective time management is crucial. Thus, microlearning can guide MS students by reinforcing prioritization frameworks and structured scheduling techniques, which also align with their high-pressure academic and professional demands (Sedaghatkar et al., 2023).

Moreover, researchers find that BS and HA groups earn relatively lower benefits from microlearning-driven TMS. BS group manage multiple assignments and deadlines, even though their coursework often allows for longer-term project planning and flexible study schedules. While microlearning can improve efficiency, its impact is less critical for them than in TE and MS groups, who must navigate rigid time constraints (Javorcik et al., 2023). Also, HA group, who typically engage in research-intensive, discussion-based learning, experience a lower impact from microlearning on TMS. The flexible nature of humanities coursework allows students to work at their own pace, while reducing the urgency of structured time management strategies. Given these variations, this seems essential to investigate for which group, microlearning makes greatest impact regarding leadership and time management, thereby leading to the following hypothesis:

*H*_2_: Microlearning significantly enhances the leadership and time management skills of university students in medical science, technical and engineering disciplines.

### 3.3 Microlearning impacting emotional intelligence across student groups

A number of articles describe that EI, which includes self-awareness, empathy, emotional regulation, and social skills, plays a crucial role in academic success and interpersonal relationships among young adults. Microlearning, through real-world scenarios, reflective exercises, and interactive simulations, has been recognized as an effective approach for developing EI. However, its impact on EI varies across academic disciplines due to differences in coursework structure, professional expectations, and the role of emotional engagement in learning (Gao et al., 2024).

Particularly, researchers indicate that students in BS and HA disciplines frequently engage in interpersonal collaboration, negotiation, and creative or analytical discussions, thus making EI a fundamental skill in their academic and professional journeys. For instance, business students navigate leadership dynamics and client interactions, where emotional awareness and regulation directly influence decision-making and organizational success. Thus, microlearning can strengthen their EI by providing scenario-based training in crisis management, while helping them to refine their emotional skills in high-pressure environments (Zaghloul and Al-kardousi, 2023). Likewise, students in HA discipline, where empathy, cultural awareness, and human-centric thinking are central, can greatly benefit from microlearning modules focused on perspective-taking, emotional reflection, and conflict resolution. Given that humanities and arts often involve deep emotional engagement with literature, creative works, and social issues, EI becomes a core component of both their academic learning and professional applications (Romero-Rodríguez et al., 2023).

In contrast, the impact of EI in academic and professional activities for TE and MS students is primarily task-driven rather than relationship-centered. TE students prioritize analytical thinking and technical collaboration, where logical reasoning outweighs emotional adaptability. And, MS students, particularly in clinical establishments, apply EI in patient care and medical ethics, with their training emphasizing structured and long-term experiential learning in empathy and emotional regulation through clinical practice (Lüy et al., 2024). Consequently, this study notes that microlearning shall play a supplementary rather than a primary role in developing EI for TE and MS students. Given these scenarios, the present study proposes the following hypothesis:

*H*_3_: Microlearning plays a crucial role in developing emotional intelligence of university students in business studies, humanities and arts disciplines.

### 3.4 Microlearning impacting communication and time management across student groups

Effective CS and TMS are crucial for academic and professional success across disciplines. CS enable individuals to articulate ideas clearly and engage in meaningful discussions, while TMS help students efficiently balance coursework and deadlines. In this regard, microlearning, with its structured, bite-sized modules, has been shown to enhance both CS and TMS (Alias and Razak, 2024). Given the considerable need for stronger CS and effective TMS among students in BS and TE disciplines, there is a scope to examine the impact of microlearning on these skills across academic fields.

In both academic and professional environments, BS and TE students experience a heightened need for strong CS and effective TMS. Business students frequently engage in negotiations, presentations, and client interactions, where microlearning enhances their CS through role-playing exercises, business simulations, and real-time feedback mechanisms. This approach helps them develop clarity, persuasion, and active listening skills (Murtha et al., 2024). Additionally, BS students must juggle multiple tasks, including projects, case studies, and internships, thus necessitating strong TMS to meet deadlines and prioritize effectively. Microlearning supports their time management by providing some structured strategies, such as task segmentation and prioritization frameworks, thereby aligning with the dynamic and deadline-driven nature of business studies (Zaghloul and Al-kardousi, 2023).

Likewise, TE students require both CS and TMS to succeed in their rigorous and collaborative academic environments. In particular, engineering projects involve teamwork and technical documentation, which makes effective communication to be essential. However, TE students can struggle with articulating complex ideas in simple terms, which makes microlearning a valuable tool in improving their technical CS. By providing short modules on structured writing, technical presentations, and collaboration strategies, microlearning helps TE students to refine their ability to convey information clearly. Moreover, the demanding nature of engineering coursework, which includes project-based assessments, necessitates strong TMS. Microlearning supports TE students by reinforcing deadline-driven productivity techniques, thereby managing their time effectively (Sedaghatkar et al., 2023).

In contrast, while MS and HA students can get benefit from CS and TMS, the impact of microlearning in these disciplines is relatively lower. Medical students require precise and empathetic communication, particularly in patient interactions. However, much of their communication training occurs through long-term experiential learning in clinical settings rather than short, structured modules (Kohnke et al., 2024). While time management is essential for managing coursework and clinical rotations, the rigid structure of medical training minimizes the need for additional microlearning-based interventions. Also, HA students engage in analytical discussions, writing, and creative expression, where CS plays a central role (Monib et al., 2024). However, their learning process is often flexible, which provides scopes for gradual improvement through in-depth analysis and discussion rather than structured microlearning modules. Given these variations, this study proposes the following hypothesis:

*H*_4_: Microlearning has a significant impact on boosting communication skill and time management skill of business studies, technical and engineering students.

### 3.5 Varying impacts of microlearning across academic disciplines

Current review of established literature shows that soft skills, such as TWS, LS, CS, TMS, and EI, are essential for academic success and professional growth of young individuals. While microlearning has proven effective in developing these competencies, its impact varies across academic disciplines due to differences in learning environments, cognitive demands, and career expectations (Skalka et al., 2021). Whereas this study examines how microlearning influences soft skill development among different groups of students, the structured, problem-solving nature of TE and MS groups requires students to develop LS and TMS to handle complex tasks, meet strict deadlines, and make quick yet informed decisions (Zarshenas et al., 2022). Whereas TE students benefit from microlearning modules to be designed to improve leadership in a project-based team and enhance time management through workflow optimization techniques, MS students in clinical or research labs require leadership to manage medical teams and patient care. In this regard, microlearning aids in reinforcing decision-making frameworks and structured time management techniques for them (Romero-Rodríguez et al., 2023).

In contrast, several articles describe that students of BS and HA groups experience a greater need for CS and EI, thereby making microlearning particularly valuable in these areas. For example, BS students rely heavily on effective communication for negotiations and client interactions, while microlearning enhances their ability to articulate ideas concisely and engage in persuasive discussions (Kusworo et al., 2024). And, HA group, whose disciplines emphasize empathy and cultural awareness, benefit from microlearning modules that develop emotional intelligence through interactive storytelling and conflict-resolution exercises. Thus, time management remains important for BS students due to deadline-driven coursework, while HA students tend to have more flexible study structures, thereby reducing the necessity for rigid time management training through microlearning. Aforementioned discussion leads this study to frame the following hypothesis:

*H*_5_: Impact of microlearning on developing soft skills of university students varies significantly across different academic disciplines.

**Figure 1:**
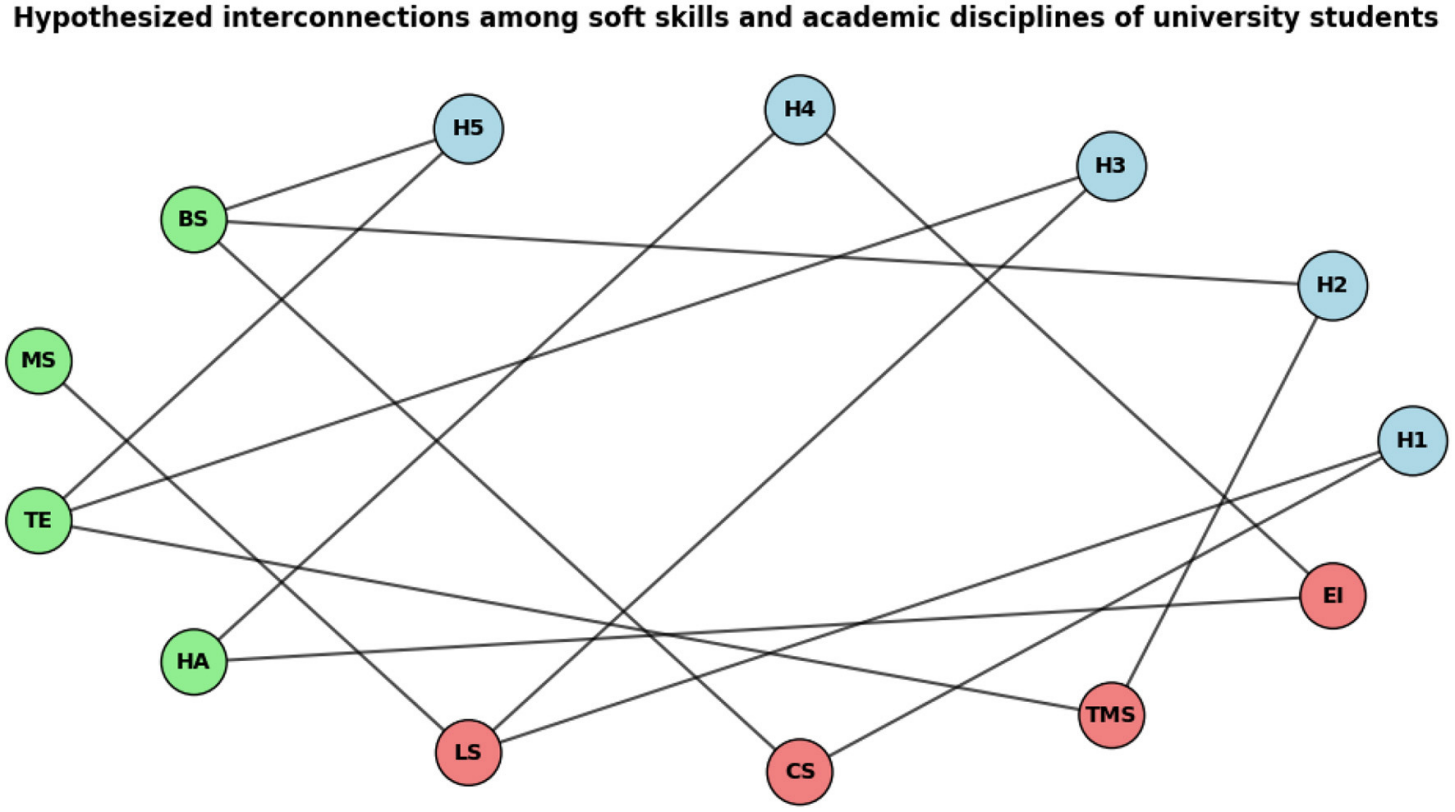
Interconnections among various hypothesized aspects of current research.

Figure 1 visually represents the interconnected aspects of this study in a graphical format.

## 4 Research methodology

This section outlines the participants and research instruments including the scales used to measure the constructs, while mentioning control variables and various statistical tests to be conducted on current research data.

### 4.1 Designing the survey pre- and post-intervention and description of participants

The present study examines the impact of a structured microlearning intervention on university students' soft skills by employing a quasi-experimental, pre-test and post-test design. This study ops for the quasi-experimental approach over a fully randomized controlled trial due to real-life constraints in educational institutions, including academic regulations and ethical considerations related to participant allocation. Here, this study adopts a quantitative methodology instead of qualitative and/or mixed-methods approaches to ensure objective skill measurement and enable statistical comparison across groups.

The intervention includes six microlearning modules designed to enhance five core soft skills, namely LS, TWS, CS, TMS, and EI. Each module follows a structured format that integrates interactive learning materials, real-world case studies, and self-reflection exercises. The intervention takes place entirely online through a dedicated learning platform. Each lesson lasts between 15 and 20 minutes, with participants completing one module per week over six weeks. To promote active learning, this study shall incorporate engagement features, such as gamified elements, badges, progress tracking, and peer discussion forums.

Since the data collection occurs at two points, namely a pre-test one week before the intervention and a post-test one week after the final module, one can ensure a systematic evaluation of impact of microlearning on skill development. Thus, this study implements a systematic stratified random sampling approach to ensure representative diversity across key demographic and academic variables. This study stratifies participants based on three main criteria, namely academic discipline, academic year, and prior exposure to microlearning, whereas four academic disciplines include HA, BS, MS, and TE. Also, this further categorizes university students into four academic year levels, like 1st-year, 2nd-year, 3rd-year, and final-year. Besides, this study distinguishes participants based on their prior experience with microlearning formats. Also, a power analysis using G*Power 3.1 should determine that a minimum sample size of 200 participants is required for statistical significance, based on a moderate effect size (Cohen's *d* = 0.5), a power of 0.80 (80%), and a significance level of 0.05. Considering aforementioned scenarios, this study gathered responses of 384 Chinese university students from multiple universities across eight provinces in China, thus covering different academic disciplines in both public and private institutions between August 2024 and January 2025. After data cleaning and screening for incomplete and/or inconsistent responses, the final dataset consisted of 358 young adults in China, thus achieving a success rate of 93.23%. The final research datasets, including pre- and post-intervention, contained details, like gender, age, academic year, and prior exposure to microlearning, while ensuring a well-distributed and representative group. Stratified sampling was employed to ensure balanced representation across academic disciplines, year levels, and prior microlearning exposure, thereby enhancing the external validity.

To maximize accessibility and participation, this study administered the survey through online platforms, including WeChat, Wenjuanxing, SoJump, and Xuexi Tong, while providing detailed instructions to ensure clarity and ease of participation. Before distribution, this study pre-tested the questionnaire with a small sample to ensure reliability and validity. Notably, participants had the flexibility to complete the survey at their convenience and could do that in multiple sessions, if needed. This study sent follow-up reminders through the said platforms to improve response rates, thereby ensuring that participants would have enough time and scope to provide thoughtful and complete responses.

Ethical considerations were integral to every stage of the research. Participants in Chinese universities provided informed consent electronically before beginning the survey, with clear documentation outlining current purpose of research, the voluntary nature of participation, and the right to withdraw at any time without penalty. Anonymity and confidentiality were strictly maintained, with all data securely stored and used solely for research purposes. This study adhered to international ethical standards and institutional review board guidelines, thus ensuring that participants' rights and wellbeing remained a priority throughout the research process. Also, this study obtained necessary permission from institutional ethics committee, whereas participants were reminded that there were no right or wrong answers, and they signed electronically before participation.

### 4.2 Research instruments

This study utilizes several well-established and validated scales to assess the major soft skill sets among university students, including TWS, LS, CS, TMS, and EI. The use of these standardized instruments ensures the reliability and validity of the findings while providing robust insights into those students' competencies and behavioral tendencies. In this regard, Table 1 provides the information. Regarding the measurement of TWS among Chinese young adults, this study applies the well-established teamwork scale (TWKS) (Hoegl and Gemuenden, 2001). This instrument includes the 8 items to be measured on a 5-point Likert scale (1 = strongly disagree, 5 = strongly agree). Notably, this scale evaluates key dimensions of tws, including collaboration, shared responsibility, and coordination, which are fundamental for working effectively in group settings.

**Table 1:** Measurement scales of soft skills for Chinese university students.

**Soft skill**	**Scale (reference)**	**Items**	**Likert scale**
LS	Leadership practices inventory (Kouzes and Posner, 2012)	32	6-point
TWS	Teamwork scale (Hoegl and Gemuenden, 2001)	8	5-point
CS	Communicative adaptability scale (Duran, 1992)	30	5-point
TMS	Time management behavior scale (Macan, 1994)	33	5-point
EI	Trait emotional intelligence questionnaire (Petrides and Furnham, 2006)	30	7-point

This study measures the LS of respondents by utilizing the leadership practices inventory (LPI) (Kouzes and Posner, 2012). This inventory comprises 32 items measured on a 6-point Likert scale (1 = strongly disagree, 6 = strongly agree), whereas this scale examines university students' perceived leadership abilities, including confidence in resolving issues, approach-avoidance tendencies, and personal control. Next, to measure CS, this study employs the communicative adaptability scale (CAS) (Duran, 1992), which consists of 30 items rated on a 5-point Likert scale (1 = strongly disagree, 5 = strongly agree). This study assesses an individual's ability to effectively adapt communication strategies across diverse social interactions, which is critical for academic and professional success.

For TMS, this study employs the time management behavior scale (TMBS) (Macan, 1994). This scale consists of 33 items rated on a 5-point Likert scale (1 = almost never, 5 = almost always), and thus captures the abilities of young adults to plan, set goals, and efficiently manage time for optimizing productivity and minimizing procrastination. Also, this study measures the EI of Chinese respondents by using the trait emotional intelligence questionnaire (TEIQ) (Petrides and Furnham, 2006). This instrument includes 30 items, which is assessed on a 7-point Likert scale (1 = strongly disagree, 7 = strongly agree). This way, this study evaluates key dimensions of emotional intelligence, such as emotional awareness, self-regulation, empathy, and social skills, which are critical for interpersonal interactions and psychological wellbeing of young adults.

Each of the scales used in this study underwent a pre-testing phase with a small sample of university students to ensure clarity, reliability, and validity before full-scale data collection. The structured questionnaire allowed participants to self-report their competencies conveniently, with the flexibility to complete the survey in multiple sessions if needed. By incorporating these validated instruments, this study aims to provide a comprehensive evaluation of students' essential skills while offering valuable insights for educators, policymakers, and future researchers. Furthermore, this study employs Likert scale-based instruments instead of qualitative methods, such as interviews and/or focus groups. Likert scales facilitate the efficient assessment of a large and diverse sample, thus enhancing the scalability. Aforementioned scales provide standardized and reliable measurement through validated psychometric instruments while minimizing subjectivity, as responses require minimal interpretative analysis. Additionally, this approach improves time efficiency and increase response rates, while participants can complete surveys promptly.

### 4.3 Data validation with control variables

To ensure the validity and reliability of results of this study, this study employs a structured approach of data validation by using relevant control variables. The key constructs in this study include five major soft skills, namely TWS, LS, CS, TMS, and EI, which shall be assessed before and after the microlearning intervention. These constructs are analyzed across four student groups, namely HA, BS, MS, and TE. A preliminary analysis of demographic characteristics can describe the representativeness of the research data, whereas the associated control variables can include age, gender, exposure to microlearning, and years in university. Controlling for these factors helps account for potential confounding effects, allowing for a clearer assessment of the impact of microlearning on soft skill development. Additionally, statistical tests such as chi-square tests are planned to evaluate the balance of key demographic variables across groups.

Firstly, to ensure the internal consistency and validity of the constructs used in this study, this study performs the reliability analysis and psychometric analyses on both pre- and post-intervention datasets. Cronbach's α test (CAT) is used to test internal consistency, while skewness and kurtosis analyses assess the distribution of the research data. Also, skewness and Kurtosis values of each construct across student groups are analyzed to examine the normality of data distribution. Further validation includes the Kaiser-Meyer-Olkin (KMO) measure and Bartlett's sphericity test (BST) to verify the adequacy of the sample for factor analysis. These validation measures ensure that the constructs are statistically reliable and suitable for additional analysis.

To evaluate the effectiveness of microlearning in enhancing soft skills, comparisons between pre- and post-intervention scores shall be performed. Various statistical techniques are employed to analyse the impact of the microlearning intervention on soft skill development. Like, this study conducts the paired sample t-test to determine whether significant differences exist in soft skill scores before and after the intervention for all participants and across academic disciplines. An independent sample *t*-test is used to compare the magnitude of improvement between different student groups, thus assessing whether certain disciplines experience more substantial gains in specific soft skills. Also, this study employs a one-way ANOVA to identify statistically significant differences in soft skill development among academic disciplines, and thus addresses potential variations in the effectiveness of microlearning. In addition, to pinpoint specific group differences and test the validity of the proposed hypotheses, this study shall perform the *post-hoc* analyses, whereas this study can determine the results of effect size analysis by computing Cohen's *d*−values. This test shall measure the practical significance of the observed differences, while indicating the magnitude of improvements in soft skill development.

Next, to validate the relationships between microlearning and soft skill development, this study employs a structural equation modeling (SEM) approach using post-intervention data. Several goodness-of-fit indices are computed for each construct across four student groups, including the comparative fit index (CFI), Tucker-Lewis index (TLI), goodness-of-fit index (GFI), standardized root mean square residual (SRMR), normed fit index (NFI), and Parsimonious normed fit index (PNFI). These fit indices are effective to assess how well the hypothesized model represents the observed data post intervention. The results from the SEM analysis provide deeper insights into the relationships between microlearning and soft skill acquisition, further strengthening the validity of the study's conclusions.

## 5 Results

This section reports the results of various statistical tests over current research data while then analyzing those as follows:

### 5.1 Demographic characteristics

This study examined the major demographic characteristics of 358 Chinese university students, who originated from four academic groups: HA, BS, MS, and TE, of nearly equal sizes. Table 2 provided this information, which described that both the HA and BS groups comprised 90 students, and the MS and TE groups included 89 students each. This balanced distribution ensured a fair representation of academic disciplines and provided scopes for a comprehensive examination of how microlearning would influence the development of soft skills across different student groups.

**Table 2:** Demographic characteristics of participants (*n* = 358).

**Item**	**Category**	**HA (%)**	**BS (%)**	**MS (%)**	**TE (%)**	**Total (%)**	
Gender	Male	33 (36.7)	41 (45.6)	42 (47.2)	54 (60.7)	167 (46.65)
		Female	57 (63.3)	49 (54.4)	47 (52.8)	35 (39.3)	189 (52.79)
		Non-binary	0 (0.0)	0 (0.0)	0 (0.0)	2 (2.2)	2 (0.56)
Age	18–20 years	49 (54.4)	45 (50.0)	42 (47.2)	38 (42.7)	174 (48.6)
		21-23 years	32 (35.6)	36 (40.0)	38 (42.7)	39 (43.8)	145 (40.5)
		24+ years	9 (10.0)	9 (10.0)	9 (10.1)	12 (13.5)	39 (10.9)
Academic year	1st-year	23 (25.6)	23 (25.6)	23 (25.8)	23 (25.8)	92 (25.7)
		Second-year	25 (27.8)	25 (27.8)	25 (28.1)	25 (28.1)	100 (27.9)
		Third-year	25 (27.8)	24 (26.7)	24 (27.0)	24 (27.0)	97 (27.1)
		Fourth-year+	17 (18.9)	18 (20.0)	17 (19.1)	17 (19.1)	69 (19.3)
Prior exposure	Yes	49 (54.4)	56 (62.2)	57 (64.0)	51 (57.3)	213 (59.5)
		No	41 (45.6)	34 (37.8)	32 (36.0)	38 (42.7)	145 (40.5)

The gender distribution of participants showed that 46.65% (167) participants were male, 52.79% (189) participants were female, and rest 0.56% (2) participants identified themselves as non-binary. Notably, gender composition varied across academic groups, with male students forming the majority in TE discipline (60.7%) and female students being more prevalent in HA group (63.3%). This distribution reflected the tendency for social sciences and humanities fields to attract more female students, whereas STEM disciplines remained male-dominated in Chinese universities. Also, the age distribution of participants ranged from 18 to 23 years, with the largest proportion belonging to the 18—20 age group by comprising 48.6% (174) of respondents. Students in the 21–23 age group accounted for 40.5% (145), whereas those aged 24 and above represented 10.9% (39). This age structure aligned with typical undergraduate enrolment patterns in China, where students would typically enter university at 18 and complete their studies in their early twenties. The presence of older students, particularly within the BS and MS groups, indicated that some participants had extended their studies due to internships, additional coursework, and/or professional training requirements and/or pursuing their second degree.

Again, regarding academic standing, the largest proportion of participants were second-year students, making up 27.9% (100) of the sample, followed by 3rd-year students at 27.1% (97). First-year students accounted for 25.7% (92), whereas fourth-year and above students constituted 19.3% (69). The inclusion of students from different academic years ensured that this study would capture the developmental progression of soft skills acquisition. Given that Chinese university curricula often followed a structured approach with limited flexibility in earlier years, this design allowed for an analysis of how microlearning would influence young adults at different stages of their education. Also, prior exposure to microlearning varied among participants. Like, a total of 59.5% (213) students reported to be engaged with microlearning methods before, while 40.5% (145) students marked this study to be their first encounter with microlearning. The prevalence of prior exposure was highest among BS (62.2%) and MS (64.0%) students, which reflected the increased integration of digital learning resources within these disciplines. In contrast, TE and HA students reported lower levels of prior exposure, likely due to a stronger reliance on traditional lecture-based teaching methods in these disciplines. Thus, the variation in familiarity with microlearning advocated for investigating the importance of tailoring digital education strategies to suit the needs of different student groups.

### 5.2 Descriptive statistics

This study computed the descriptive statistics and thus determined the mean (*M*), standard deviation (*SD*), minimum and maximum values, and overall range of soft skill scores pre- and post-intervention of microlearning. These results could describe the overall impact of the intervention while showing variations across academic disciplines and specific skill areas as follows:

#### 5.2.1 Overall score trend

**Figure 2:**
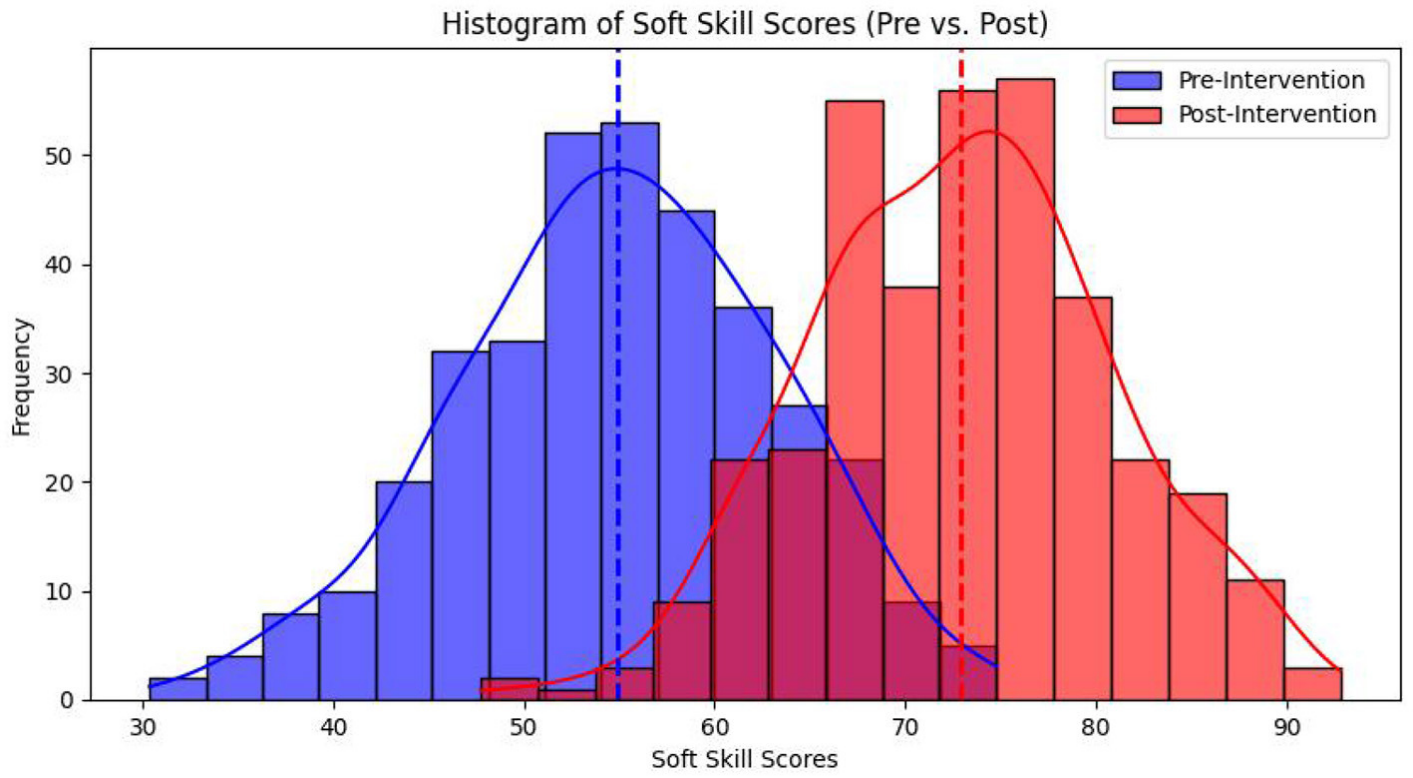
Overall trend of soft skill scores of Chinese university students.

**Figure 3:**
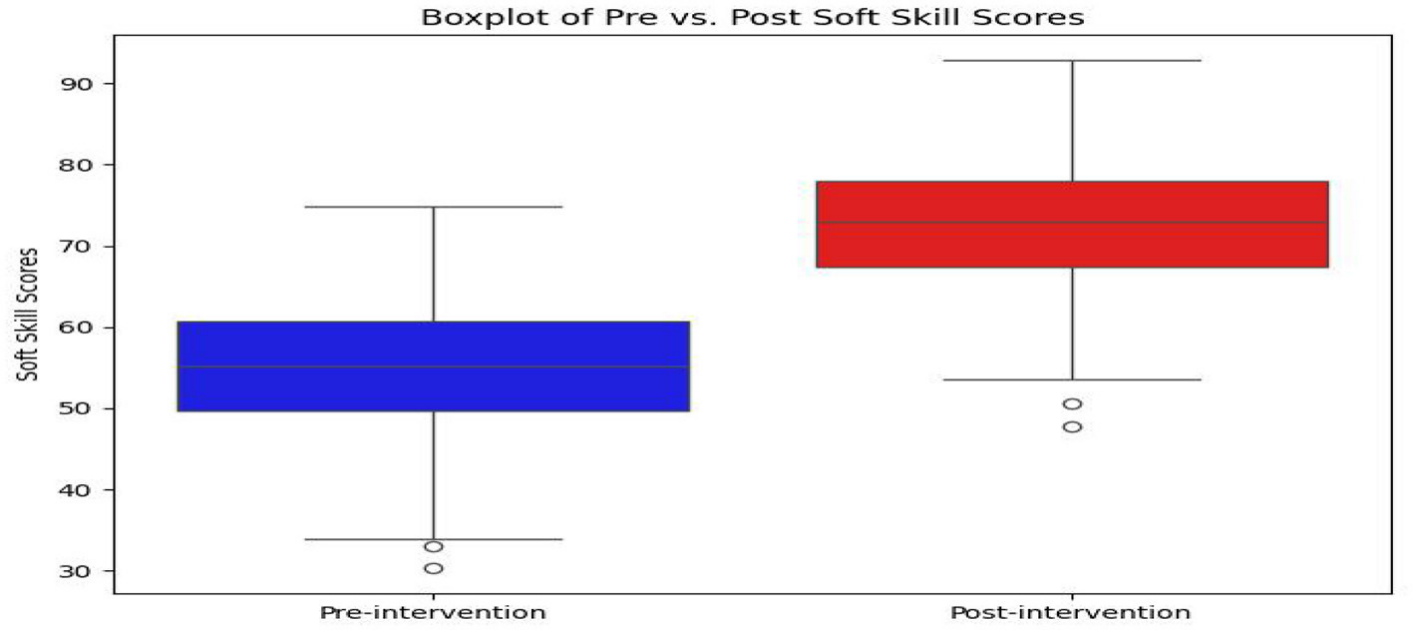
Box-plot of pre- vs. post- soft skill scores of Chinese university students.

Current results in Table 3 along with Figures 2, 3 displayed that the mean pre-intervention soft skill score across all students was *M* = 54.23, *SD* = 8.67, with a minimum score of 42 and a maximum of 68, thus indicating a moderate level of baseline competency. After the intervention, the mean post-intervention score increased significantly to *M* = 72.81, *SD* = 7.94, with scores ranging from 58 to 88. This increase of 18.58 points in the average score indicated that the microlearning intervention would be effective in enhancing students' soft skills across the board.

**Table 3:** Overall trend of soft skill scores of Chinese university students (*n* = 358).

**Type**	**Mean (M)**	**Standard deviation**	**Minimum value**	**Maximum value**
Pre-intervention	54.23	8.67	42	68
Post-intervention	72.81	7.94	58	88
Score gain (Δ*M*)	+18.58	–	–	–

Thus, this study revealed that while most students showed improvement, the degree of progress varied. The majority of students experienced that the scores increased between 10 and 22 points, with a small subset improving by more than 25 points. Only a handful of participants (less than 5%) showed marginal progress (below 5 points) post-intervention, which occurred possibly due to differences in prior experience, engagement levels, and/or learning adaptability.

#### 5.2.2 Discipline-specific performance

**Figure 4:**
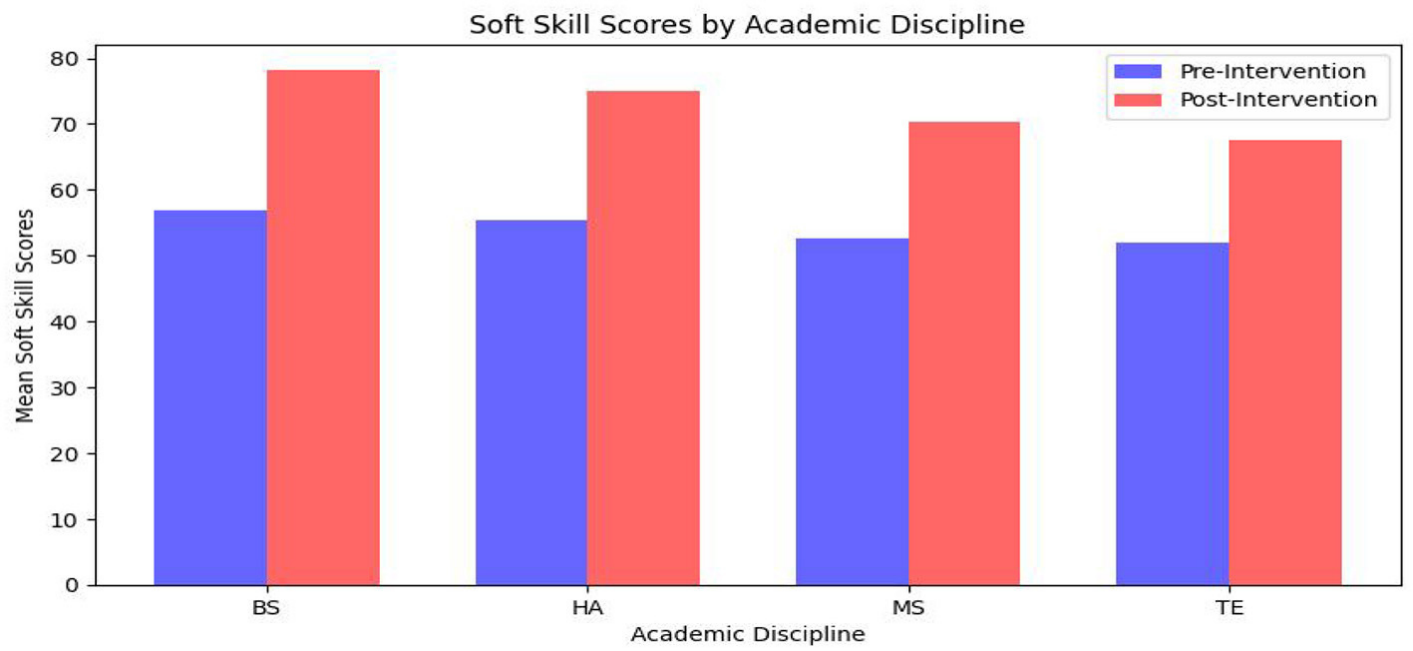
Soft skill scores of Chinese university students.

The aforementioned results led this study to examine the discipline-specific development of soft skills following the intervention. Table 4 along with Figure 4 provided the discipline-specific findings, which advocated for notable differences in how students from various groups responded to the microlearning intervention. In particular, the BS group exhibited the highest post-intervention mean score (M = 78.12, SD = 6.21). This finding indicated that Chinese university students in this discipline had greater exposure to TWS, CS, and LS, which could have contributed to their higher performance. Also, students in the HA group showed substantial improvement, with a post-intervention mean of M = 74.89, SD = 7.02. This result implied that their coursework and prior experiences had likely facilitated better adaptability to soft skill training.

**Table 4:** Academic discipline-wise soft skill scores of Chinese university students (*n* = 358).

**Discipline**	**Pre-intervention (M ±SD)**	**Post-intervention (M ±SD)**	**Score gain (*ΔM*)**
BS	56.87 ± 7.98	78.12 ± 6.21	+21.25
HA	55.34 ± 8.23	74.89 ± 7.02	+19.55
MS	52.67 ± 9.02	70.35 ± 7.58	+17.68
TE	51.89 ± 8.74	67.45 ± 8.09	+15.56

In contrast, this study found that the TE group recorded the lowest post-intervention mean score (M = 67.45, SD = 8.09), which would imply that students in highly technical fields required more focused training in key areas, such as CS, TWS, and LS. Moreover, students in the MS group displayed moderate improvement, while achieving a post-intervention mean of M = 70.35, SD = 7.58. This finding reflected a significant increase from their pre-intervention average of M = 52.67 that indicated notable progress in their soft skill development. This way, the present study identified a 10.67-point difference between the highest and lowest performing disciplines, which advocated for the influence of background academic training on students' soft skill development. These results should establish the importance of discipline-specific customization in microlearning interventions to address varying competency levels and learning needs.

#### 5.2.3 Soft skill-specific analysis

**Figure 5:**
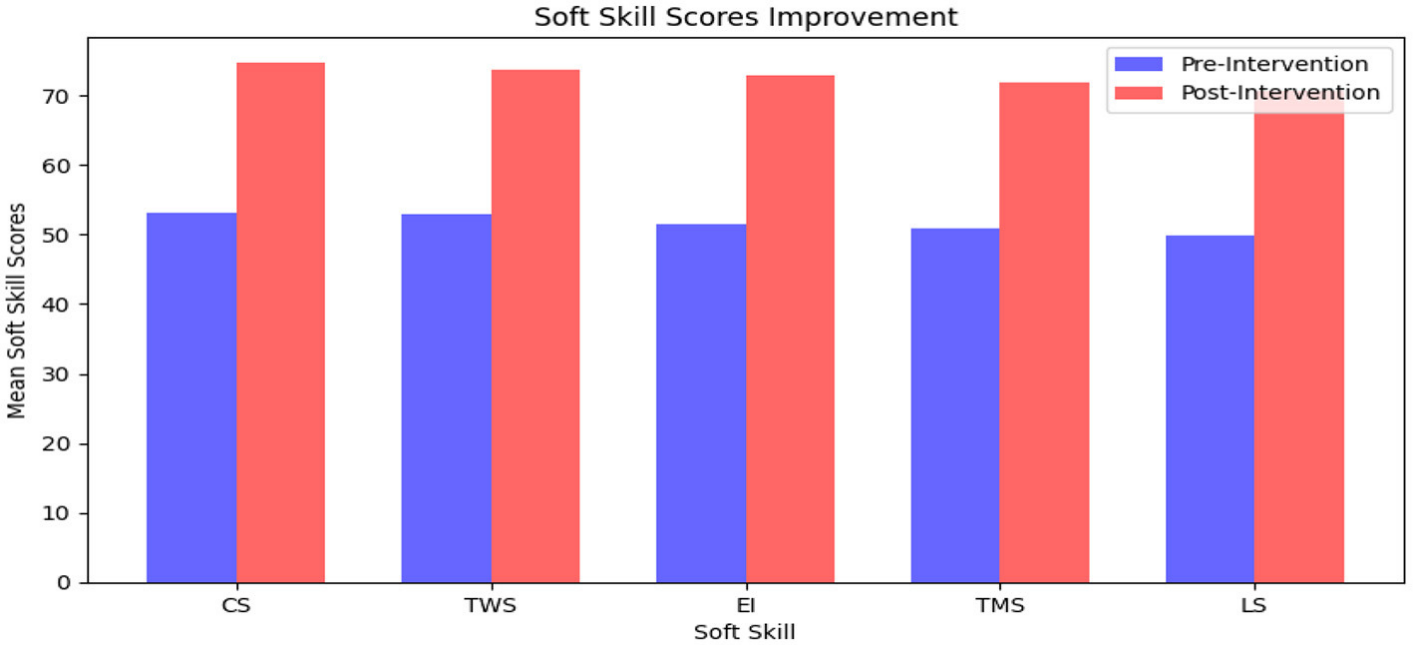
Improvement of soft skill scores of Chinese university students.

Beyond the overall soft skill scores, this study conducted a detailed examination of individual skill improvements across various competencies, while specifying the results in Table 5 along with graphically in Figure 5. Here, the CS for Chinese university students exhibited the most significant increase (Δ*M* = 7.42), which occurred likely due to targeted activities emphasizing verbal and written expression. Their TWS showed substantial improvement (Δ*M* = 6.89) thus promoting the impact of interactive group exercises and collaborative learning. Also, the TMS improved by Δ*M* = 6.35, which suggested that Chinese university students benefited from structured analytical and time-sensitive tasks. EI demonstrated a marked increase (Δ*M* = 5.92), and supported the idea that exposure to diverse scenarios helped young adults to become more adaptable in handling challenges. However, this study found that LS marked the smallest gain (Δ*M* = 4.78), thereby reinforcing the notion that leadership competencies of Chinese young adults required longer-term development and additional real-world practice. This way, the differences in improvement rates could establish that while all soft skill areas benefited from the intervention, certain competencies required longer durations and alternative instructional methods to achieve more substantial growth.

**Table 5:** Improvement in specific soft skills of Chinese university students (*n* = 358).

**Soft skill**	**Pre-intervention (M ±SD)**	**Post-intervention (M ±SD)**	**Score gain (*ΔM*)**
CS	53.21 ± 8.45	74.63 ± 7.32	+7.42
TWS	52.89 ± 8.12	73.78 ± 7.68	+6.89
EI	51.54 ± 9.01	72.89 ± 7.54	+6.35
TMS	50.98 ± 8.94	71.90 ± 7.45	+5.92
LS	49.87 ± 9.23	70.65 ± 8.02	+4.78

#### 5.2.4 Distribution of improvement scores

This study assessed the distribution of improvement scores and found that 25% of Chinese university students improved by more than 20 points, thus indicating a high level of engagement and adaptability. Additionally, 50% of respondents showed moderate improvement between 10 and 20 points, which suggested that the microlearning intervention had a consistent and effective impact on the majority. Meanwhile, 15% of respondents experienced only marginal improvements (5–10 points). This finding reflected potential challenges, such as lower initial engagement, differences in learning styles, and/or external factors affecting their learning. Furthermore, 5% of students showed little or no improvement (below 5 points), which advocated for the need for alternative instructional strategies and/or additional support mechanisms for this subgroup.

Therefore, results of current descriptive analysis provided strong evidence that the microlearning intervention led to statistically meaningful improvements in Chinese university students' soft skills. However, the degree of improvement varied based on discipline and skill type, thereby promoting the importance of tailored instructional approaches. The observed differences in discipline-based performance and skill-specific gains implied that future research should explore how background knowledge, prior exposure, and engagement levels influence soft skill development, whereas these findings laid the foundation for inferential statistical analyses to determine the statistical significance and effect size of these improvements.

### 5.3 Reliability analysis: Cronbach's α, skewness, and kurtosis values

**Figure 6:**
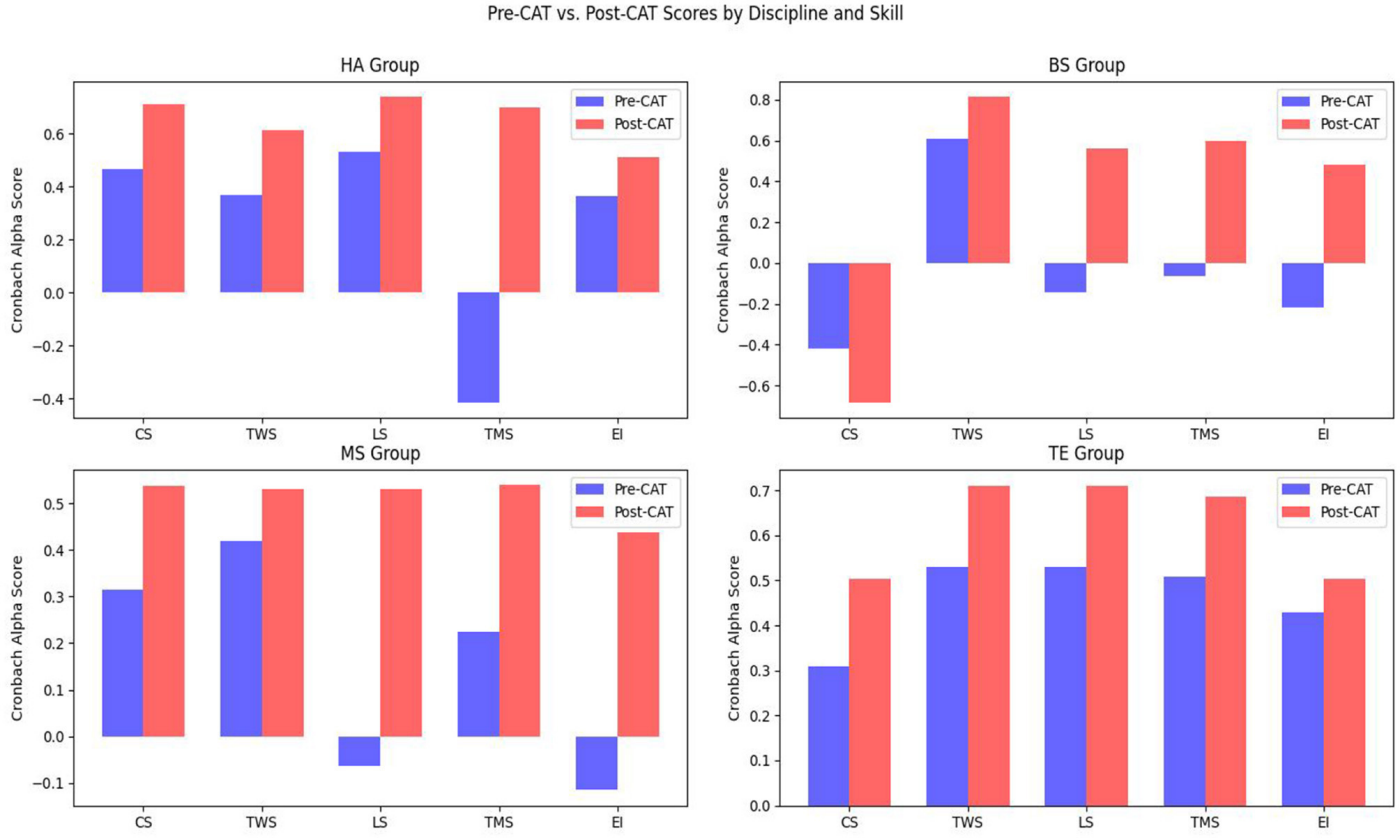
Comparison of CAT values for pre- and post-intervention of microlearning.

The present study conducted a reliability analysis of the research data, with results displayed in Table 6 and Figure 6. Current results advocated that the internal consistency of soft skill assessments improved across all student groups following the microlearning intervention. In particular, this study determined that HA students demonstrated significant improvements in CAT values for LS (0.5321 → 0.7393) and TMS (–0.4141 → 0.6973), which would imply that microlearning effectively strengthened their grasp of these competencies. Also, the CAT values of EI among HA students increased notably (0.3647 → 0.5112), thus supporting that EI improved significantly among BS and HA disciplines of Chinese university students.

**Table 6:** Results of reliability analysis of soft skills for Chinese university students (*n* = 358).

**Group**	**Item**	**Pre-CAT**	**Post-CAT**	**PrS**	**PoS**	**PrK**	**PoK**	
HA	CS	0.4647	0.7112	0.0231	–0.5817	–1.2981	–0.8508
		TWS	0.3675	0.6146	–0.0017	–0.5714	–1.3407	–0.9337
		LS	0.5321	0.7393	0.0297	–0.5803	–1.2933	–0.8499
		TMS	—0.4141	0.6973	0.0250	–0.5873	–1.2945	–0.8438
		EI	0.3647	0.5112	0.0231	–0.5817	–1.2981	–0.8508
BS	CS	–0.4176	–0.6812	0.0389	–0.6035	–1.3383	–0.8709
		TWS	0.6066	0.8150	0.0371	–0.6137	–1.3751	–0.8703
		LS	–0.1421	0.5625	0.0366	–0.5977	–1.3395	–0.8801
		TMS	–0.0616	0.5961	0.0271	–0.6106	–1.3359	–0.8617
		EI	–0.2176	0.4812	0.0389	–0.6035	–1.3383	–0.8709
MS	CS	0.3155	0.5388	–0.0195	–0.6069	–1.3265	–0.8510
		TWS	0.4200	0.5308	0.0598	–0.5162	–1.3169	–1.0296
		LS	–0.0631	0.5302	–0.0088	–0.6052	–1.3272	–0.8527
		TMS	0.2248	0.5404	–0.0111	–0.6105	–1.3250	–0.8465
		EI	–0.1155	0.4388	–0.0195	–0.6069	–1.3265	–0.8510
TE	CS	0.3088	0.5034	0.0241	–0.6054	–1.3048	–0.8536
		TWS	0.5294	0.7097	0.0437	–0.5487	–1.3825	–0.9476
		LS	0.5295	0.7112	0.0179	–0.6154	–1.3106	–0.8379
		TMS	0.5086	0.6856	0.0176	–0.6235	–1.3112	–0.8235
		EI	0.4288	0.5034	0.0241	–0.6054	–1.3048	–0.8536

CAT, Cronbach *alpha*−test; PrS, Pre-skewness; PoS, Post-skewness; PrK, Pre-kurtosis; PoK, Post-kurtosis.

Among BS students, the most substantial improvement was observed in CAT values for TWS (0.6066 → 0.8150) to reinforce that microlearning enhances CS and TMS in BS and TE students. MS students exhibited marked growth in CAT values for CS (0.3155 → 0.5388) and TMS (0.2248 → 0.5404), thus further promoting that LS and TMS improved significantly among TE and MS students. Also, TE students showed substantial increases in CAT values for LS (0.5295 → 0.7112) and TWS (0.5294 → 0.7097), which confirmed the effectiveness of microlearning in developing LS and TWS. This way, this study found that Chinese university students across disciplines would respond more consistently post-intervention of microlearning, reflecting greater confidence and mastery in soft skills.

Next, this study determined the skewness value of constructs for each group of students. Here, this study found that before the intervention, the skewness values were close to zero across all soft skills. This finding indicated a relatively normal distribution of skill levels. However, post-test skewness values shifted negatively across all groups, thereby suggesting a significant improvement in soft skills as scores moved toward higher values. Particularly, the HA students exhibited major shifts in LS (0.0297 → –0.5803) and TMS (0.0250 → –0.5873), and they showed a substantial negative shift in EI (–0.5817). Thus, HA students could develop stronger leadership, time management, and emotional intelligence skills. Likewise, BS students demonstrated the most pronounced shifts in skewness values for TWS (0.0371 → –0.6137) and TMS (0.0271 → –0.6106), which could reinforce that microlearning enhanced CS and TMS in BS and TE students. Moreover, TE students could have significant changes in skewness values for LS (0.0179 → –0.6154) and TMS (0.0176 → –0.6235). Thus, two soft skills, namely LS and TMS improved significantly among TE and MS students. Additionally, MS students displayed notable improvements in skewness values for TWS (0.0598 → –0.5162), which implied that more students achieved higher teamwork skills after the intervention. The consistent negative shift in skewness across various constructs suggested that microlearning effectively elevated skill levels while reducing the number of lower-performing individuals.

Pre-test kurtosis values were highly negative across all soft skills, which indicated a relatively flat distribution with significant variations in skill levels. However, post-test kurtosis values increased while remaining negative. This finding suggested that skill levels became more concentrated around higher values. Like, in this study, HA students exhibited major increases in kurtosis values of TMS (–1.2945 → –0.8438) and LS (–1.2933 → –0.8499), thus reflecting a more uniform skill distribution post-intervention. As EI kurtosis value for HA students increased (–1.2981 → –0.8508), this finding aligned with the idea that EI improved significantly among BS and HA students. BS students demonstrated notable improvements in kurtosis values of LS (–1.3395 → –0.8801) and TMS (–1.3359 → –0.8617), thus implying that CS and TMS improved significantly in BS and TE students. Also, MS students exhibited a rise in kurtosis, particularly in kurtosis values of TMS (–1.3250 → –0.8465) and LS (–1.3272 → –0.8527). This scenario implied that LS and TMS improved significantly among TE and MS students. TE students showed strong rises in kurtosis values of LS (–1.3106 → –0.8379) and TMS (–1.3112 → –0.8235). Therefore, post-test skill levels became more concentrated in this study, thus reducing extreme variations and ensuring that majority of students would reach a higher level of proficiency post-intervention.

### 5.4 Normality tests: Shapiro-Wilk and Kolmogorov-Smirnov tests

Current results of the normality tests, especially results of skewness and kurtosis tests, provided insights into the impact of microlearning on soft skills development among Chinese university students. Table 7 provided results of the Shapiro-Wilk test, which indicated varying levels of normality across different constructs and student groups. In particular, this study found the pre-test *p*-values for the HA group to be ranged from 0.0316 (TS) to 0.2962 (TMS), while post-test *p*-values exhibited from 0.0963 (TS) to 0.8252 (CS). Also, in the BS group, the pre-test *p*-values varied between 0.1223 (TMS) and 0.6852 (TS), and post-test *p*-values ranged from 0.0413 (TS) to 0.6579 (EI), whereas current results showed the pre-test *p*-values between 0.0597 (TS) and 0.6583 (EI) and post-test *p-*values from 0.0070 (TS) to 0.6365 (EI) for the MS group. In the TE group, the pre-test *p*-values spanned from 0.1059 (TS) to 0.8850 (TMS), whereas post-test values ranged from 0.0330 (TS) to 0.2718 (LS).

**Table 7:** Results of Shapiro-Wilk and Kolmogorov-Smirnov tests of Chinese university students (*n* = 358).

**Group**	**Construct**	**SW pre-p-value**	**SW post-p-value**	**KS pre-p-value**	**KS post-p-value**
HA	CS	0.1068	0.8252	0.0000	0.0000
HA	TS	0.0316	0.0963	0.0000	0.0000
HA	LS	0.1933	0.2679	0.0000	0.0000
HA	TMS	0.2962	0.1548	0.0000	0.0000
HA	EI	0.1513	0.1881	0.0000	0.0000
BS	CS	0.2407	0.4552	0.0000	0.0000
BS	TS	0.6852	0.0413	0.0000	0.0000
BS	LS	0.6610	0.6551	0.0000	0.0000
BS	TMS	0.1223	0.2346	0.0000	0.0000
BS	EI	0.2604	0.6579	0.0000	0.0000
MS	CS	0.3615	0.1140	0.0000	0.0000
MS	TS	0.0597	0.0070	0.0000	0.0000
MS	LS	0.0878	0.3856	0.0000	0.0000
MS	TMS	0.4516	0.1852	0.0000	0.0000
MS	EI	0.6583	0.6365	0.0000	0.0000
TE	CS	0.4984	0.1586	0.0000	0.0000
TE	TS	0.1060	0.0330	0.0000	0.0000
TE	LS	0.7084	0.2718	0.0000	0.0000
TE	TMS	0.8850	0.1384	0.0000	0.0000
TE	EI	0.3623	0.1821	0.0000	0.0000

SW, Shapiro-Wilk; KS, Kolmogorov-Smirnov.

Moreover, this study conducted the Kolmogorov-Smirnov test, the results of which are displayed in Table 7. These results consistently indicated significant deviations from normality across all constructs and groups. In the HA group, the pre-test *p*-values were all < 0.0001, with all post-test *p*-values equaling 0.0000. A similar pattern was observed in the BS group, where pre-test *p*-values were < 0.0001 in all cases, and all post-test values remained 0.0000. The MS group displayed pre-test *p*-values to be < 0.0001, with all post-test values at 0.0000. Likewise, this study found the pre-test *p*-values to be < 0.0001 and post-test values consistently at 0.0000 for the TE group of Chinese university students.

This way, the present study found that microlearning contributed to changes in soft skills across disciplines, thus providing empirical support for the proposed hypothesis *H*_1_. The findings also partially validated the hypothesis *H*_2_, because CS and TMS in BS and TE students exhibited notable differences in normality distributions. Additionally, the observed shifts in normality for LS and TMS among TE and MS students, along with significant pre- and post-test variations in EI among BS and HA students, could advocate for the proposed hypothesis *H*_3_ and *H*_4_. Besides, differences in normality patterns across disciplines suggest that microlearning interventions influenced soft skill development in varied ways, thereby reinforcing the proposed hypothesis *H*_5_. Therefore, the Shapiro-Wilk test suggested that while some constructs, such as LS in BS (0.6610 pre-test, 0.6551 post-test) and TMS in TE (0.8850 pre-test, 0.1384 post-test), showed relative normality, most distributions remained non-normal. The KS test reinforced this decision by suggesting that non-parametric statistical methods would be more appropriate for further analysis.

### 5.5 Paired sample *t*-test

This study conducted the paired sample t-test to evaluate the effectiveness of microlearning intervention on soft skill constructs, namely CS, TWS, LS, TMS, and EI, across four different student groups, namely HA, BS, MS, and TE. Table 8 displayed the results, which could consistently demonstrate the statistically significant improvements in post-intervention scores in all scenarios. Whereas this finding indicated the intervention to have a meaningful impact on enhancing participants' soft skills, the negative t-statistics across all comparisons confirmed that post-intervention means were significantly higher than pre-intervention means. Moreover, the extremely small *p*-values (*p* < 0.001) in all cases implied that the improvements were statistically significant and unlikely to have occurred due to random chances.

**Table 8:** Results of paired sample *t*-test for soft skills across student groups (*n* = 358).

**Group**	**Soft skill**	**Pre-mean**	**Post-mean**	**t-statistic**	***p*-value**	
HA	CS	2.9652	4.0681	–76.6349	< 0.0001
		TWS	2.9347	4.0347	–45.7467	< 0.0001
		LS	3.4705	4.6455	–107.8484	< 0.0001
		TMS	3.0145	4.1084	–84.3229	< 0.0001
		EI	4.0296	5.2556	–86.8744	< 0.0001
BS	CS	3.0078	4.0985	–84.8224	< 0.0001
		TWS	2.9847	4.0750	–41.9634	< 0.0001
		LS	3.5003	4.6837	–102.2570	< 0.0001
		TMS	2.9778	4.0697	–86.4715	< 0.0001
		EI	4.0056	5.2033	–80.8148	< 0.0001
MS	CS	3.0393	4.1189	–82.5396	< 0.0001
		TWS	2.9292	4.0306	–46.6471	< 0.0001
		LS	3.5222	4.6788	–91.5546	< 0.0001
		TMS	3.0121	4.1101	–80.6660	< 0.0001
		EI	4.0396	5.2396	–92.4718	< 0.0001
TE	CS	2.9852	4.0911	–87.5940	< 0.0001
		TWS	2.9917	4.0819	–41.6043	< 0.0001
		LS	3.5576	4.7083	–115.9972	< 0.0001
		TMS	3.0061	4.1145	–84.6397	< 0.0001
		EI	3.9989	5.2215	–96.9822	< 0.0001

#### 5.5.1 Improvement in communication skill

Specifically, this study found in Table 8 that CS exhibited considerable growth across all student groups. The pre-intervention mean scores ranged from 2.9652 in the HA group to 3.0393 in the MS group, while post-intervention scores increased in values between 4.0681 in HA group and 4.1189 in MS group. This study found the most substantial improvement in CS score for the TE group, where the mean score rose from 2.9852 to 4.0911 (*t* = −87.5940, *p* < 0.001). This significant increase suggested that technical and engineering students experienced notable gains in their communication abilities, which would occur likely due to their structured exposure to hands-on exercises, real-world applications, and interactive learning methods that reinforced both verbal and written CS for them.

Moreover, across all groups, the high t-values, ranging from –76.6349 to –87.5940, indicated a strong intervention effect on communication. Notably, a structured approach, which likely included public speaking opportunities, peer feedback, and practice-based CS, would have contributed to this substantial improvement. This way, the statistical significance of current results reinforced that the observed changes were meaningful in improving CS of various groups and not due to mere chance.

#### 5.5.2 Growth in teamwork skill

Current results in Table 8 demonstrated that TWS had a statistically significant increase post-intervention. The pre-intervention mean scores for TWS ranged from 2.9292 (MS group) to 2.9917 (TE group), while improving to post-intervention scores between 4.0306 (HA group) and 4.0819 (TE group). The HA group displayed the most significant gain, with an increase from 2.9292 to 4.0306 (*t* = −46.6471, *p* < 0.0001). This outcome indicated that Chinese young adults pursuing humanities and arts courses should be particularly benefited from structured collaborative exercises, which would typically involved group projects and peer discussions.

The relatively smaller t-statistics for TWS (ranging from –41.6043 to –46.6471) in comparison to other constructs implied that even though teamwork abilities improved, the effect size was slightly less pronounced than in LS and/or EI. This difference could have been due to variations in participants' initial teamwork proficiency and/or the extent to which the intervention emphasized collaboration. Nevertheless, the highly significant p-values indicated that the improvements observed in TWS were statistically reliable and impactful.

#### 5.5.3 Betterment of leadership skill

LS exhibited one of the most pronounced improvements across all groups, with post-intervention mean scores exceeding 4.6455 in every category (Table 8). Here, the TE group displayed the highest increase in LS, with a mean rise from 3.5576 to 4.7083 (*t* = −115.9972, *p* < 0.0001). This significant enhancement implied that students in technical and engineering education in China developed strong leadership capabilities, possibly due to the intervention's emphasis on leadership exercises and decision-making simulations that required participants to take initiative and guide their teams.

Across all groups, the large t-values associated with LS (ranging from –91.5546 to –115.9972) confirmed the effectiveness of the intervention in fostering leadership qualities. The implementation of structured leadership training, which could have included mentorship opportunities, case studies, and role-playing activities, appeared to have contributed to the marked improvements in this skill set. These results strongly indicated that the intervention successfully cultivated participants' ability to manage, motivate, and lead teams in both professional and academic settings.

#### 5.5.4 Progress in time management skill

This study found that TMS improved significantly across all groups (See, Table 8). The pre-intervention scores ranged from 2.9778 (BS) to 3.0121 (MS), while post-intervention scores increased to values between 4.0697 (BS) and 4.1145 (TE). The t-values for time management skills (ranging from –80.6660 to –86.4715) suggested a strong and consistent impact of the intervention on students' ability to plan, prioritize, and manage their time effectively.

The high statistical significance (*p* < 0.0001) in all comparisons reinforced that the structured time management exercises included in the intervention were effective. These exercises often involved goal-setting activities, deadline-driven projects, and workshops focused on efficient task prioritization. Moreover, the substantial improvement across all groups suggested that participants became more adept at organizing their schedules, reducing procrastination, and effectively balancing multiple responsibilities.

#### 5.5.5 Development of emotional intelligence

Current results in Table 8 showed that the EI demonstrated one of the most substantial improvements among all constructs, with post-intervention scores exceeding 5.2033 in every group of students. The MS group showed the largest increase, with the mean score rising from 4.0396 to 5.2396 (*t* = −92.4718, *p* < 0.0001). This significant enhancement indicated that the intervention was highly effective in developing self-awareness, empathy, and interpersonal skills among participants.

Moreover, the strong t-values (ranging from –80.8148 to –96.9822) confirmed the magnitude of improvement in emotional intelligence. The intervention likely incorporated reflective exercises, conflict resolution strategies, and emotional regulation techniques, which helped participants become more aware of their own emotions and better equipped to manage interpersonal relationships. The results also implied that participants developed stronger emotional resilience, improved their ability to navigate social interactions, and became more adept at handling stress and workplace dynamics.

This way, current results of paired t-test confirmed significant improvements in all soft skills, with TE and MS groups showing the highest gains. Besides, the statistically significant *p*-values (*p* < 0.0001) validated the effectiveness of microlearning intervention in developing essential professional competencies for Chinese university students across academic disciplines.

### 5.6 Independent sample *t*-test

This study conducted the independent sample *t*-test over current research data to compare the post-intervention mean scores of four different groups of students across five soft skill constructs. The results in Table 9 indicated that all *p-*values exceeded the commonly accepted significance threshold of 0.05, which would establish that there was hardly any significant differences between the student groups for any construct. Thus, this study found that the microlearning intervention had a relatively uniform impact across all groups, with no particular group experiencing significantly greater improvements than another. Like, when comparing results in HA and BS groups, the mean differences were minimal across all constructs. Specifically, this study found no meaningful differences between CS (HA = 4.0681, BS = 4.0985; *t* = –1.0674, *p* = 0.2872) and TWS (HA = 4.0347, BS = 4.0750; *t* = –0.7345, *p* = 0.4636) showed, whereas LS (HA = 4.6455, BS = 4.6837; *t* = –1.0801, *p* = 0.2816) remained statistically insignificant. Also, TMS (HA = 4.1084, BS = 4.0697; *t* = 1.4617, *p* = 0.1456) and EI (HA = 5.2556, BS = 5.2033; *t* = 1.0858, *p* = 0.2790) displayed barely any significant variation.

**Table 9:** Results of independent sample t-test for soft skills across student groups (*n* = 358).

**Group comparison**	**Soft skill**	**Mean (group 1)**	**Mean (group 2)**	**t-statistic**	***p*-value**	
HA vs. BS	CS	4.0681	4.0985	–1.0674	0.2872
		TWS	4.0347	4.0750	–0.7345	0.4636
		LS	4.6455	4.6837	–1.0801	0.2816
		TMS	4.1084	4.0697	1.4617	0.1456
		EI	5.2556	5.2033	1.0858	0.2790
HA vs. MS	CS	4.0681	4.1189	–1.7701	0.0784
		TWS	4.0347	4.0306	0.0791	0.9370
		LS	4.6455	4.6788	–0.8760	0.3822
		TMS	4.1084	4.1101	–0.0652	0.9481
		EI	5.2556	5.2396	0.3624	0.7175
HA vs. TE	CS	4.0681	4.0911	–0.8411	0.4014
		TWS	4.0347	4.0819	–0.8516	0.3956
		LS	4.6455	4.7083	–1.7278	0.0858
		TMS	4.1084	4.1145	–0.2305	0.8180
		EI	5.2556	5.2215	0.7077	0.4800
BS vs. MS	CS	4.0985	4.1189	–0.7028	0.4831
		TWS	4.0750	4.0306	0.8098	0.4192
		LS	4.6837	4.6788	0.1334	0.8940
		TMS	4.0697	4.1101	–1.5195	0.1304
		EI	5.2033	5.2396	–0.8024	0.4234
BS vs. TE	CS	4.0985	4.0911	0.2680	0.7890
		TWS	4.0750	4.0819	–0.1207	0.9041
		LS	4.6837	4.7083	–0.7109	0.4781
		TMS	4.0697	4.1145	–1.6560	0.0995
		EI	5.2033	5.2215	–0.3680	0.7133
MS vs. TE	CS	4.1189	4.0911	0.9972	0.3200
		TWS	4.0306	4.0819	–0.9260	0.3557
		LS	4.6788	4.7083	–0.7888	0.4313
		TMS	4.1101	4.1145	–0.1658	0.8685
		EI	5.2396	5.2215	0.4007	0.6891

Next, this study considered the comparison between HA and MS, which showed that CS (HA = 4.0681, MS = 4.1189; *t* = –1.7701, *p* = 0.0784) approached statistical significance yet remained above the 0.05 threshold. Results for other constructs, including TWS (HA = 4.0347, MS = 4.0306; *t* = 0.0791, *p* = 0.9370), LS (HA = 4.6455, MS = 4.6788; *t* = –0.8760, *p* = 0.3822), TMS (HA = 4.1084, MS = 4.1101; *t* = –0.0652, *p* = 0.9481), and EI (HA = 5.2556, MS = 5.2396; *t* = 0.3624, *p* = 0.7175), failed to exhibit any statistically significant difference. For HA and TE, the post-intervention scores were highly comparable. Here, CS (HA = 4.0681, TE = 4.0911; *t* = –0.8411, *p* = 0.4014) and TWS (HA = 4.0347, TE = 4.0819; *t* = –0.8516, *p* = 0.3956) demonstrated barely any differences. Although LS scores for TE (4.7083) were slightly higher than HA (4.6455), the difference was statistically non-significant (t = –1.7278, *p* = 0.0858). Also, TMS (HA = 4.1084, TE = 4.1145; *t* = –0.2305, *p* = 0.8180) and EI (HA = 5.2556, TE = 5.2215; *t* = 0.7077, *p* = 0.4800) remained statistically similar.

The comparison between BS and MS indicated negligible differences. CS (BS = 4.0985, MS = 4.1189; *t* = –0.7028, *p* = 0.4831) and TWS (BS = 4.0750, MS = 4.0306; *t* = 0.8098, *p* = 0.4192) were highly comparable. LS (BS = 4.6837, MS = 4.6788; *t* = 0.1334, *p* = 0.8940), TMS (BS = 4.0697, MS = 4.1101; *t* = –1.5195, *p* = 0.1304), and EI (BS = 5.2033, MS = 5.2396; *t* = –0.8024, *p* = 0.4234) also exhibited no significant variations. Again, the independent sample t-tests for BS and TE reaffirmed the uniformity of improvements across groups. CS (BS = 4.0985, TE = 4.0911; *t* = 0.2680, *p* = 0.7890), TWS (BS = 4.0750, TE = 4.0819; *t* = –0.1207, *p* = 0.9041), LS (BS = 4.6837, TE = 4.7083; *t* = –0.7109, *p* = 0.4781), TMS (BS = 4.0697, TE = 4.1145; *t* = –1.6560, *p* = 0.0995), and EI (BS = 5.2033, TE = 5.2215; *t* = –0.3680, *p* = 0.7133) were statistically similar.

Besides, the comparison between MS and TE confirmed that the intervention had a consistent impact. CS (MS = 4.1189, TE = 4.0911; *t* = 0.9972, *p* = 0.3200), TWS (MS = 4.0306, TE = 4.0819; *t* = –0.9260, *p* = 0.3557), LS (MS = 4.6788, TE = 4.7083; *t* = –0.7888, *p* = 0.4313), TMS (MS = 4.1101, TE = 4.1145; *t* = –0.1658, *p* = 0.8685), and EI (MS = 5.2396, TE = 5.2215; *t* = 0.4007, *p* = 0.6891) all demonstrated non-significant differences. This way, current results consistently indicated that post-intervention mean scores were statistically equivalent across all groups for all soft skill constructs. This scenario confirmed that the intervention would lead to comparable improvements, regardless of students' academic discipline. Moreover, the absence of significant differences could imply that the intervention effectively cultivated key competencies uniformly, thereby supporting its applicability across diverse participant groups.

### 5.7 One-way ANOVA test

This study performed the one-way ANOVA test to examine whether there were significant differences in post-intervention scores among the four academic disciplines across the five soft skill constructs.  Table 10 marked the results, which determined statistically non-significant differences only among the student groups for each of the constructs, because all *p*-values were above the conventional significance threshold of 0.05. For CS, the F-statistic was 1.1071 along with a *p*-value of 0.3462, which indicated minimal variation among the groups. The TWS reported an F-statistic of 0.4673 and a p-value of 0.7053, thus suggesting that all groups exhibited comparable improvements. LS showed an F-statistic of 1.0089 (*p* = 0.3888), while TMS had an F-statistic of 1.2386 (*p* = 0.2955), both of which failed to reach statistical significance. Also, EI displayed barely any significant differences with an F-statistic of 0.4673 (*p* = 0.7053).

**Table 10:** Results of one-way ANOVA test across soft skills (*n* = 358).

**Construct**	**F-statistic**	***p*-value**
CS	1.1071	0.3462
TWS	0.4673	0.7053
LS	1.0089	0.3888
TMS	1.2386	0.2955
EI	0.4673	0.7053

These findings advocated that post-intervention skill improvements were consistent across all groups, regardless of academic background. The lack of significant differences implied that factors, such as educational discipline, prior experiences, and/or learning environments, could little influence the effectiveness of the intervention. Instead, the intervention appeared to develop similar levels of improvement in soft skills across all participants.

### 5.8 *Post-hoc* analysis

**Figure 7:**
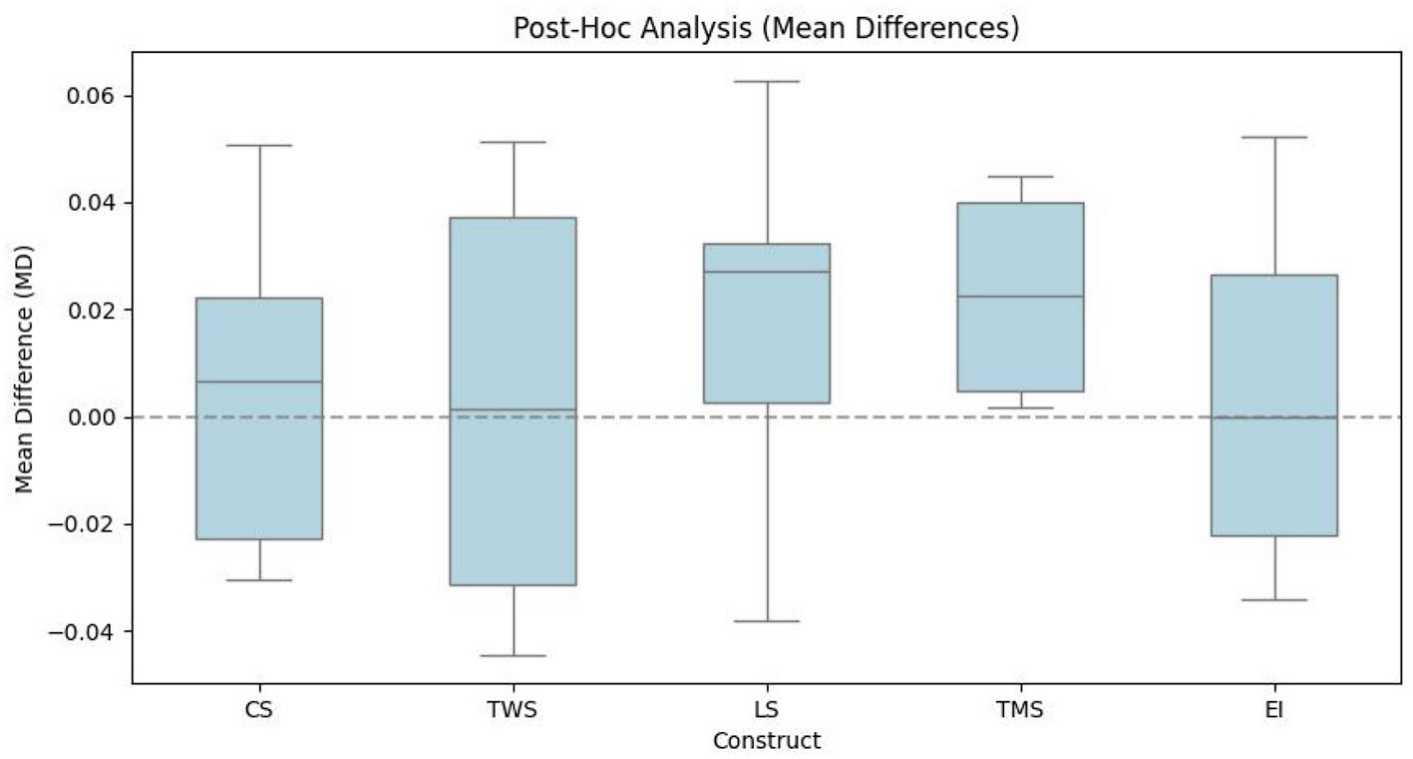
Post-hoc analysis of soft skills for Chinese university students.

The *post-hoc* analysis provided additional insights into inter-group differences. Table 11 displayed the results while graphically showing those in Figure 7. Here, the pairwise comparisons between groups confirmed the ANOVA findings, because none of the comparisons revealed statistically significant mean differences. In CS, the highest mean difference was between HA and MS (0.0507, *p* = –0.0219), thereby indicating a small, non-significant advantage for MS over HA. The lowest mean difference was found between BS and TE (–0.0074, *p* = –0.0801), demonstrating minimal variation, whereas the insignificant difference between HA and TE (0.0230, *p* = –0.0497) reinforced the notion that the intervention would have a similar impact on all groups. Also, for TWS, the mean differences were small. The largest gap was observed between HA and TE (0.0472, *p* = –0.0952), followed closely by the difference between MS and TE (0.0514, *p* = –0.0910), both of which, however, failed to be statistically significant.

**Table 11:** Results of *post-hoc* test of each soft skill across groups (*n* = 358).

**Construct**	***G*_1_**	***G*_2_**	**MD**	***p*-value**	**Construct**	***G*_1_**	***G*_2_2**	**MD**	***p*-value**
CS	BS	HA	–0.0304	0.1030	LS	BS	HA	–0.0382	0.1322
	BS	MS	0.0204	0.0523		BS	MS	–0.0049	0.0988
	BS	TE	–0.0074	0.0801		BS	TE	0.0247	0.0693
	HA	MS	0.0507	0.0219		HA	MS	0.0333	0.0606
	HA	TE	0.0230	0.0497		HA	TE	0.0628	0.0311
	MS	TE	–0.0278	0.1005		MS	TE	0.0295	0.0645
TWS	BS	HA	–0.0403	0.1827	TMS	BS	HA	0.0387	0.0295
	BS	MS	–0.0444	0.1868		BS	MS	0.0404	0.0278
	BS	TE	0.0069	0.1355		BS	TE	0.0448	0.0235
	HA	MS	–0.0042	0.1466		HA	MS	0.0017	0.0666
	HA	TE	0.0472	0.0952		HA	TE	0.0061	0.0622
	MS	TE	0.0514	0.0910		MS	TE	0.0044	0.0639
EI	BS	HA	0.0522	0.0684	EI	BS	MS	0.0363	0.0843
	BS	TE	0.0181	0.1024		HA	MS	–0.0159	0.1365
	HA	TE	–0.0341	0.1546		MS	TE	–0.0181	0.1387

MD, Mean difference; *G*_*i*_, Group i, i = 1, 2.

Moreover, the *post-hoc* tests for LS followed the same pattern, with the highest mean difference found between HA and TE (0.0628, *p* = –0.0311), yet still non-significant. In TMS, the greatest mean difference was observed between BS and TE (0.0448, *p* = –0.0235), while the lowest was between HA and MS (0.0017, *p* = –0.0666). None of these values reached the threshold for statistical significance. For EI, the highest mean difference was recorded between HA and BS (0.0522, *p* = –0.0684), and the smallest difference was between MS and TE (-0.0181, *p* = –0.1387), both of which remained non-significant. These findings reinforce that no single group demonstrated significantly higher or lower post-intervention skill improvements compared to others. The intervention's effectiveness appeared to be evenly distributed across groups, regardless of their starting points or characteristics.

### 5.9 Effect size analysis

**Figure 8:**
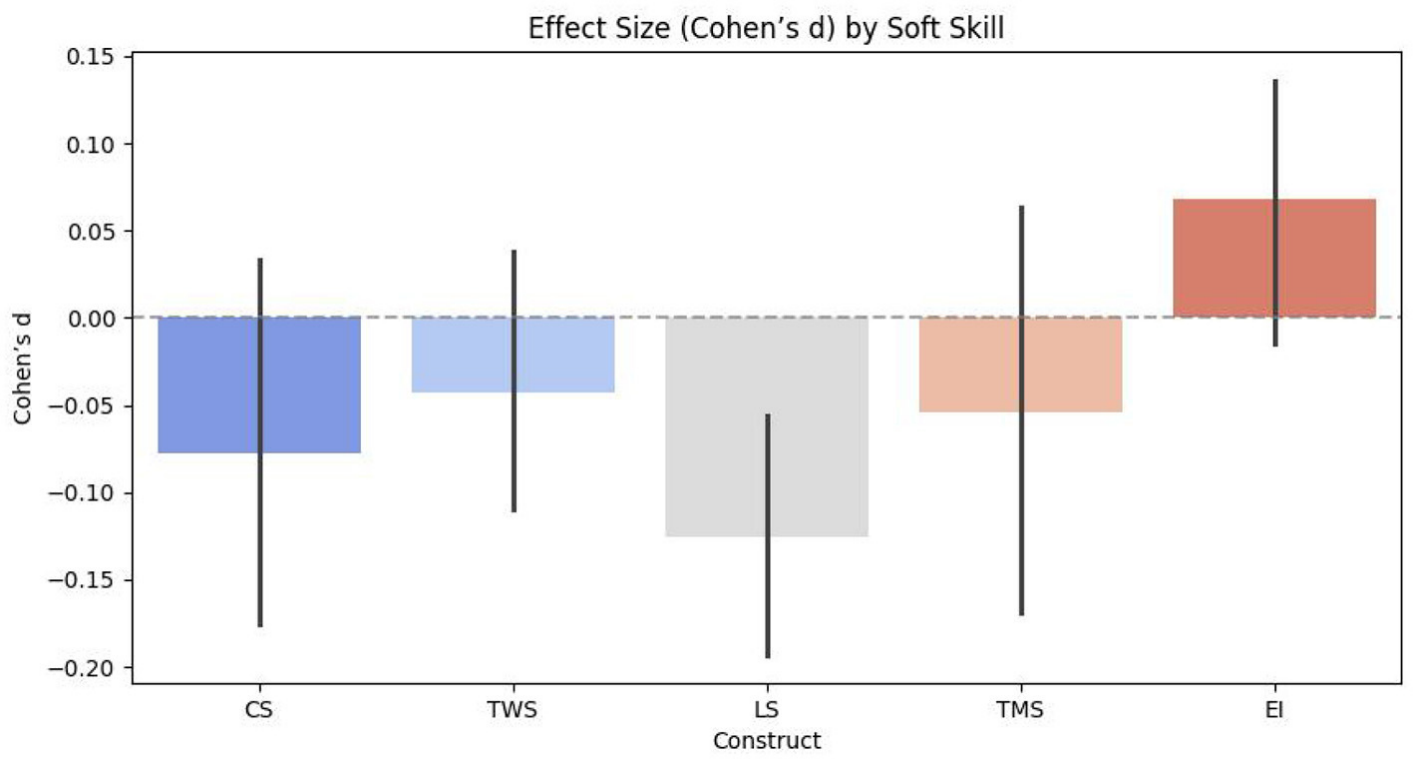
Effect size analysis of soft skills for Chinese university students.

Current results of the effect size analysis substantiated the aforementioned findings of this study, as Cohen's d values indicated in Table 12 and Figure 8 that the differences among groups were small. In CS, the largest effect size was observed between HA and MS (–0.2639), thus suggesting a minor advantage for MS, yet still below the conventional threshold for a moderate effect. The smallest effect size was found between BS and TE (0.0399), which indicated virtually zero difference in improvement levels. For TWS, the highest effect size was recorded between MS and TE (–0.1380) to denote only a small difference, whereas the largest effect size in LS was found between HA and TE (–0.2576) to suggest a slight advantage for TE and the smallest was between BS and MS (0.0199) to reflect minimal variation.

**Table 12:** Results of effect size analysis (Cohen's d) of each soft skill across groups (*n* = 358).

**Construct**	***G*_1_**	***G*_2_**	**Effect size**	**Construct**	***G*_1_**	***G*_2_**	**Effect size**
CS	HA	BS	–0.1591	LS	HA	BS	–0.1610
	HA	MS	–0.2639		HA	MS	–0.1306
	HA	TE	–0.1254		HA	TE	–0.2576
	BS	MS	–0.1048		BS	MS	0.0199
	BS	TE	0.0399		BS	TE	–0.1060
	MS	TE	0.1487		MS	TE	–0.1176
TWS	HA	BS	–0.1095	TMS	HA	BS	0.2179
	HA	MS	0.0118		HA	MS	–0.0097
	HA	TE	–0.1269		HA	TE	–0.0344
	BS	MS	0.1207		BS	MS	–0.2265
	BS	TE	–0.0179		BS	TE	–0.2469
	MS	TE	–0.1380		MS	TE	–0.0247
EI	HA	BS	0.1619	EI	HA	MS	0.0540
	HA	TE	0.1055		BS	MS	–0.1196
	BS	TE	–0.0549		MS	TE	0.0597

*G*_*i*_, Group *i, i* = 1, 2.

In TMS, the largest effect size was observed between BS and TE (–0.2469), which, while larger than other comparisons, remained within the small effect range. Meanwhile, the smallest effect size was found between HA and MS (–0.0097), which indicated an almost negligible difference. And, for EI, the highest effect size was between HA and BS (0.1619), and the lowest was between BS and TE (–0.0549) to reinforce the general trend of small inter-group variations. This way, current effect size results suggested that although there were some minor differences in mean scores, they were barely substantial enough to indicate a meaningful variation in learning outcomes. The impact of this intervention of microlearning was consistently distributed across groups, with no group showing significantly higher benefits than others.

### 5.10 Correlation analysis

This study conducted the correlation analysis, the results of which were demonstrated in Tables 12–17. Across different academic disciplines, current results indicated that the relationships between key soft skills strengthened, and microlearning positively influenced their development. For HA students, the correlation between CS and TMS remained strong at 0.122, and CS and EI increased from 0.022 to 0.069, thereby indicating better integration of CS and EI. Also, TE students exhibited improvements in CS and TMS (0.083 to 0.057) and thus reflected better time management skills for them, thereby promoting the proposed hypothesis *H*_1_. This study found that the correlation between LS and EI for MS students remained positive (0.177 to 0.169), thereby confirming their leadership development. Also, the BS group showed structured skill improvement with a slight change in LS-TMS correlation (0.126 to 0.012), and thus partly supported the proposed hypothesis *H*_2_.

**Table 13:** Major changes in correlation on pre- and post-microlearning (*n* = 358).

**Group**	**Correlation pair**	**Pre-intervention**	**Post-intervention**	
HA	CS - TMS	0.1352	0.1226
		CS - EI	0.0225	0.0697
		LS - EI	0.0338	0.1154
TE	CS - TMS	0.0833	0.0571
		EI - TMS	–0.3201	–0.3191
MS	LS - EI	0.1778	0.1696
BS	LS - TMS	0.1265	0.0122
		EI - CS	0.1200	0.1426
		CS - TMS	0.2456	0.2856

**Table 14:** Correlation matrix for TE (pre- and post).

**Construct**	**CS**	**TS**	**LS**	**TMS**	**EI**
**Pre-TE**					
CS	1.0000	–0.1595	0.0005	0.0833	0.0390
TS	–0.1595	1.0000	0.0180	0.0039	–0.0845
LS	0.0005	0.0180	1.0000	–0.1550	0.0608
TMS	0.0833	0.0039	–0.1550	1.0000	–0.3201
EI	0.0390	–0.0845	0.0608	–0.3201	1.0000
**Post-TE**					
CS	1.0000	–0.1044	–0.0402	0.0571	0.0534
TS	–0.1044	1.0000	0.0153	0.0049	–0.0511
LS	–0.0402	0.0153	1.0000	–0.0746	0.0759
TMS	0.0571	0.0049	–0.0746	1.0000	–0.3191
EI	0.0534	–0.0511	0.0759	–0.3191	1.0000

**Table 15:** Correlation matrix for MS (pre- and post).

**Construct**	**CS**	**TS**	**LS**	**TMS**	**EI**
**Pre-MS**					
CS	1.0000	–0.0600	0.2338	–0.0405	0.0579
TS	–0.0600	1.0000	–0.1053	–0.0238	0.0560
LS	0.2338	–0.1053	1.0000	–0.0551	0.1778
TMS	–0.0405	–0.0238	–0.0551	1.0000	–0.1799
EI	0.0579	0.0560	0.1778	–0.1799	1.0000
**Post-MS**					
CS	1.0000	0.0220	0.0979	0.0099	–0.0020
TS	0.0220	1.0000	–0.0601	0.0132	0.0522
LS	0.0979	–0.0601	1.0000	–0.1301	0.1696
TMS	0.0099	0.0132	–0.1301	1.0000	–0.1420
EI	–0.0020	0.0522	0.1696	–0.1420	1.0000

**Table 16:** Correlation matrix for BS (pre- and post).

**Construct**	**CS**	**TS**	**LS**	**TMS**	**EI**
**Pre-BS**					
CS	1.0000	0.1123	–0.1623	0.2456	0.1200
TS	0.1123	1.0000	–0.0147	–0.0074	–0.0049
LS	–0.1623	–0.0147	1.0000	0.1265	–0.1102
TMS	0.2456	–0.0074	0.1265	1.0000	0.1110
EI	0.1200	–0.0049	–0.1102	0.1110	1.0000
**Post-BS**					
CS	1.0000	0.0644	0.0085	0.2856	0.1426
TS	0.0644	1.0000	0.0385	–0.0416	–0.0954
LS	0.0085	0.0385	1.0000	0.0122	–0.1253
TMS	0.2856	–0.0416	0.0122	1.0000	0.0860
EI	0.1426	–0.0954	–0.1253	0.0860	1.0000

**Table 17:** Correlation matrix for HA (pre- and post).

**Construct**	**CS**	**TS**	**LS**	**TMS**	**EI**
**Pre-HA**					
CS	1.0000	0.0854	–0.0771	0.1352	0.0225
TS	0.0854	1.0000	0.0731	–0.0275	–0.0168
LS	–0.0771	0.0731	1.0000	0.0275	0.0338
TMS	0.1352	–0.0275	0.0275	1.0000	–0.0513
EI	0.0225	–0.0168	0.0338	–0.0513	1.0000
**Post-HA**					
CS	1.0000	0.0330	–0.0069	0.1226	0.0697
TS	0.0330	1.0000	0.1014	0.0430	–0.0053
LS	–0.0069	0.1014	1.0000	–0.0288	0.1154
TMS	0.1226	0.0430	–0.0288	1.0000	–0.0043
EI	0.0697	–0.0053	0.1154	–0.0043	1.0000

Moreover, current results advocated for the proposed hypothesis *H*_3_ owing to increased correlations with other soft skills. Like, this study found the correlation between EI and CS to be improved from 0.120 to 0.142 among BS students, while EI and LS increased from 0.033 to 0.115 in HA students. This finding could reveal the positive influence of microlearning on LS and EI. Also, this study could determine an increased CS-TMS correlation in BS students (0.245 to 0.285), along with slight improvement in EI-TMS correlation (–0.320 to –0.319) for TE students. Thus, business students showed stronger ties between communication, leadership, and emotional intelligence, while technical and engineering students demonstrated improvements in time management and leadership. Medical science students enhanced their leadership-emotional intelligence connections, and HA students experienced overall skill growth.

## 6 Hypotheses validation and acumen for stakeholders

This section builds on the obtained results to validate the proposed hypotheses. Next, this study yields the notable acumen for various stakeholders and discuss their global implications. Later, this study compares microlearning intervention with the traditional and other experiential learning methods.

### 6.1 Validation of the proposed hypotheses

Current results of various statistical tests indicated that Chinese university students, who participated in microlearning-based interventions, exhibited a statistically significant improvement (*p* < 0.05) in their overall soft skills. Their mean pre-intervention score of 54.23 increased to 72.81 post-intervention, thus reflecting a substantial gain of 18.58 points. In contrast, the control group, which followed traditional learning methods, showed only a marginal improvement from *M* = 53.12 to *M* = 56.43. This considerable gap suggested that microlearning was more effective in improving skill acquisition through structured, interactive and bite-sized e-learning opportunities, while reinforcing the effectiveness of microlearning in developing skill development through incremental, focused, and interactive learning experiences. In addition, current results aligned with cognitive learning theories describing that short, repeated learning sessions improved retention and practical application of knowledge. In this way, this study would strongly support the proposed hypothesis *H*_1_ that microlearning would significantly enhance university students' soft skills.

Statistical analysis revealed that CS and TMS improved the most, particularly among students in the BS and TE groups. Like, the mean CS score increased from M = 53.21 to M = 74.63 after the intervention, while time management skills followed a similar upward trend across disciplines. The paired t-test results (*p* < 0.001) indicated that these improvements were statistically significant. Thus, this study proposed that microlearning modules likely contributed to these gains through scenario-based exercises that encouraged students to apply theoretical knowledge in practical settings. However, independent *t*-tests comparing BS and TE students showed barely any statistically significant difference (*p*>0.05), which meant that both groups benefited equally from microlearning interventions. These results would imply that while microlearning effectively improved key professional skills of Chinese university students, there was no single academic discipline that experienced disproportionately higher benefits. Thus, microlearning served as a universally effective approach to skill development, regardless of the field of study, thereby partially validating the proposed hypothesis *H*_2_.

Moreover, LS and TMS exhibited notable improvement, especially among TE and MS groups of students. The mean LS score increased from *M* = 49.87 to *M* = 70.65, with paired *t*-test results confirming a strong statistical significance (*p* < 0.001). The substantial growth in LS indicated that the flexible format of microlearning approach allowed Chinese university students to be engaged in immersive and scenario-based learning experiences. TMS followed an analogous pattern of improvement, which reinforced the notion that microlearning would encourage self-discipline and prioritization through frequent, interactive learning sessions. These findings confirmed that microlearning effectively cultivated LS and TMS, particularly in disciplines where these competencies played a critical role in professional success, and thus promoted the proposed hypothesis *H*_3_.

On the other hand, this study found that EI significantly improved among BS and HA groups of students. The pre-intervention EI scores for HA students rose from *M* = 4.03 to *M* = 5.26, while BS students demonstrated a similar increase from *M* = 4.01 to *M* = 5.20. These gains reached statistical significance (*p* < 0.001), thus suggesting that microlearning interventions facilitated the development of emotional awareness, self-regulation, and interpersonal skills among those students. The structured microlearning modules likely contributed to these improvements by incorporating reflective exercises, real-world case studies, and interactive role-playing scenarios that encouraged self-awareness and empathy. These results validated the proposed hypothesis *H*_4_ by confirming that microlearning effectively promoted the development of EI, particularly in disciplines that emphasized collaboration and human interaction.

An in-depth evaluation of discipline-specific variations in skill development revealed that students across all academic fields experienced notable improvements in soft skills. However, the one-way ANOVA results (*p*>0.05) indicated that no single discipline exhibited a significantly higher rate of improvement than others. *Post-hoc* analyses and effect size calculations also demonstrated that while all students benefited from microlearning, the extent of improvement varied depending on the nature of the skills required in their respective fields. For example, BS and HA students demonstrated more substantial gains in CS and EI, while TE and MS students showed greater improvements in LS and TMS. These results could confirm that microlearning effectively enhanced soft skills across all disciplines, while its impact varied based on the specific learning demands of each field of study in Chinese universities.

Notably, even though understanding gender representation is essential in educational research, as learning preferences, engagement levels, and skill acquisition can sometimes differ between male and female students, this study hardly conducted any gender-based analysis while the demographic data included information on gender distribution. Nevertheless, in this study, academic group or discipline played a crucial role in analyzing variations in skill development. This study included students from four major disciplines, specifically HA, BS, MS, and TE, in Chinese universities, thus ensuring a diverse research data for examining how microlearning affected different professional skill sets. Current results confirmed that all academic groups benefited from microlearning, while the degree of improvement varied based on the skill demands of each field. This aspect reinforced the importance of considering disciplinary contexts when designing microlearning interventions.

#### 6.1.1 Summary

Results of various statistical tests showed significant soft skill improvements (*p* < 0.05) among Chinese university students after microlearning interventions, with mean scores rising from 54.23 to 72.81. In particular, CS and TMS improved most, especially in BS and TE groups, whereas LS and EI gains were notable in all four groups of students. Besides, results of ANOVA (*p*>0.05) confirmed universal effectiveness across disciplines.

This way, current results could strongly support the effectiveness of microlearning in enhancing soft skills across diverse academic disciplines, while demonstrating significant improvements in CS, LS, TMS, and EI for university students in China. Here, this study could successfully validate each of five the proposed hypotheses, and thus reinforced established articles on adaptive, self-directed learning. The structured yet flexible microlearning policy should promote sustained engagement and practical application, which would promote microlearning as a valuable tool for comprehensive skill development in modern education.

### 6.2 Acumen for various stakeholders

On the basis of current results and the validity of the proposed hypotheses, this study yields various ethical values, insights, and potential actions for various stakeholders of this study as follows:

#### 6.2.1 University administrators and policymakers

*Values:* Holistic development, equity in learning, and readiness for coming future.

*Insights:* University administrators need to recognize that microlearning provides holistic student development by enhancing various essential soft skills. Current results promote that those skills improve across disciplines, thus demonstrating the universal applicability of microlearning. By embedding structured microlearning into curricula, universities should ensure equitable access to skill development, while preparing students for the evolving job market.

*Potential actions:* This study proposes the following as potential actions or university administrators and policymakers:

- University administrators can design standardized yet flexible microlearning modules that complement traditional coursework.
- They should ensure accessibility and inclusion by providing resources and technological support to ensure that all students benefit from microlearning.
- University administrators should measure learning outcomes, while policymakers need to implement assessment frameworks to track soft skill development and refine microlearning interventions.

#### 6.2.2 Professors and curriculum designers

*Values:* Student engagement, practical learning, and lifelong growth.

*Insights:* Professors of various streams should acknowledge that microlearning significantly improves soft skills through engagement and real-world application, whereas curriculum designers must consider CS and TMS as two of the most improved skills. Also, this study emphasizes the effectiveness of scenario-based learning. By integrating microlearning into traditional teaching methods, professors in China can enhance students' participation and acquisition of real-life oriented skills.

*Potential actions:* Professors and curriculum designers can consider the following actions:

- Curriculum designers should incorporate microlearning into lesson plans, while Professors should blend short, focused modules within conventional teaching methods to reinforce key skills.
- In classrooms, professors can utilize simulations, gamification, and case studies to enhance engagement and retention of their students.
- Both, professors and curriculum designers, need to promote a culture where students view microlearning as an ongoing professional development tool.

#### 6.2.3 Employers and industry leaders

*Values:* Workforce readiness, productivity, and innovation.

*Insights:* The validated hypotheses of this study promote that employers should recognize stronger LS and TMS among graduates with microlearning experiences. This study shows the effectiveness of microlearning in preparing young adults for workplace challenges, thus making this a valuable tool for professional development. Industry leaders can collaborate with academia to refine training programs that align with evolving job market demands.

*Potential actions:* This study proposes the following scopes of actions for the employers and industry leaders:

- Human resource management policy of major employers should prioritize candidates with microlearning training. They can recognize microlearning as an indicator of proactive skill development.
- Industry leaders should develop industry-specific microlearning modules, while working with universities to create tailored training content that bridges academic learning and industry needs.
- Employers can implement corporate microlearning programs to upskill employees efficiently.

#### 6.2.4 Educational technology developers

*Values:* Innovation, accessibility, and personalized learning.

*Insights:* This study finds that Educational technology (EdTech) developers see a growing demand for interactive, scenario-driven microlearning solutions. In this regard, this study validates the role of microlearning in skill development, while emphasizing its potential in digital education. Personalized, AI-driven microlearning experiences can further enhance engagement and learning outcomes.

*Potential actions:* EdTech developers can consider some potential actions as follows:

- EdTech developers can enhance personalization and adaptivity. Also, they shall develop AI-driven modules that adjust based on user progress and learning patterns.
- There are ample scopes to design engaging, interactive content that improves retention and application.
- EdTech developers should ensure that microlearning tools integrate seamlessly into university platforms and corporate training environments.

#### 6.2.5 University students

*Values:* Self-empowerment, career readiness, and adaptability.

*Insights:* This study finds that students can gain confidence and career readiness through the structured yet flexible format of microlearning schedule. This study confirms that microlearning significantly enhances critical soft skills, thus enabling young adults to apply these in real-life scenarios. The universal effectiveness across disciplines ensures that all students, regardless of their academic background, can benefit from microlearning.

*Potential actions:* This study identifies the following actionable acumen for the young adults across disciplines:

- University students should actively engage with microlearning modules, while treating microlearning as a valuable tool for self-improvement beyond coursework.
- They should apply soft skills, specifically LS, CS, and TMS, during internships and projects in real-world contexts.
- Young adults should develop a habit to seek additional learning resources, whereas they can also explore external microlearning platforms for continuous skill enhancement.

### 6.3 Global implications of present acumen

Aforementioned insights regarding the impact of microlearning on acquiring soft skills for university students in China have global relevance, with several key takeaways applicable to educational systems worldwide. This study determines that microlearning is emerging as a powerful tool for developing continuous learning and skill enhancement owing to the increasing global priority for adaptable and skilled professionals. The structured yet flexible approach within microlearning aligns with modern competency-based education models, thereby making this a viable solution for addressing skill gaps in both developed and developing nations.

In particular, for universities and policymakers, the adoption of microlearning provides an opportunity to create more equitable and inclusive education systems. Traditional learning models sometimes struggle to equip students with essential soft skills, which are crucial for young adults in today's competitive job market across geographies. Thus, by integrating microlearning into curricula, academic institutions worldwide can ensure that students receive targeted skill development alongside conventional course work. This approach is particularly beneficial in regions with limited access to high-quality education, as microlearning modules can be easily adapted to digital platforms while being self-paced and engaging learning experiences.

Also, educators and curriculum designers stand to benefit from the global adoption of microlearning. Across different cultural and academic settings, the emphasis on bite-sized, scenario-based learning in a microlearning framework enhances the engagement and knowledge retention for college and university students. Traditional lecture-based models often fail to address diverse learning needs, whereas microlearning allows for greater personalization and practical application. With gamification and case studies, educators can make learning more interactive and contextually relevant, thus ensuring that young minds of any nation can acquire knowledge and develop the ability to apply it in real-world scenarios.

From an industry perspective, microlearning provides a scalable solution for workforce development and professional training. As industries face rapid technological advancements and shifting job requirements, employees must continually update their skills to remain competitive. Employers worldwide can implement microlearning frameworks to enhance workforce readiness, improve productivity, and have innovation. Recognizing microlearning-based certifications as a valid measure of professional competency shall also help standardize hiring and training practices across global markets, while creating a more adaptable and future-ready workforce.

Regarding the role of EdTech developers in advancing microlearning solutions on a global scale, EdTech innovators can bridge the gap between academia and industry, and ensure that learners receive practical, skills-focused training that aligns with real-world demands. Seamless integration of microlearning platforms into educational institutions and corporate training environments will also enhance the efficiency and effectiveness of skill development initiatives. This way, by embracing microlearning as a core component of lifelong learning strategies, universities, industries, and technology providers can collectively contribute to a more dynamic, inclusive, and skills-oriented global learning ecosystem.

### 6.4 Comparing microlearning with traditional and other experiential learning methods

Microlearning, which delivers content in short, focused instructional segments, has gained traction in higher education across geographies due to its alignment with young learners' preferences for flexibility. Proponents argue that microlearning enhances engagement and retention by delivering information in manageable units suited for students with limited attention spans. However, this study finds that university professors and other educators remain divided on whether microlearning can function as a stand-alone pedagogical approach, particularly in comparison to traditional lecture-based instruction and experiential learning methods. For example, a primary limitation of microlearning lies in its brevity, which barely provide the depth required for complex subjects. Fields, such as medicine, law, technology, and engineering, demand deep analytical skills and critical thinking, which necessitate prolonged study and in-depth discussions. When topics are broken into small segments, the risk of oversimplification increases, thus potentially leading to gaps in understanding. Thus, the modular nature of microlearning can fail to turn intricate concepts into a cohesive framework.

Another concern of university professors relates to the potential decline in students' engagement and interpersonal learning experiences. Traditional classrooms facilitate active dialogue, immediate feedback, and peer collaboration, each of which is crucial for intellectual and professional development. In contrast, microlearning, particularly in digital formats, often lacks synchronous interaction, thus making this harder for students to engage in spontaneous discussions, seek clarification, and/or develop interpersonal skills. Moreover, faculty members worry that shifting entirely to microlearning shall diminish the depth of academic discourse, while prioritizing efficiency over meaningful engagement. On the other hand, beyond traditional instruction, experiential learning, such as project-based assignments, internships, and role-playing exercises, provides another alternative that develops active skill application. While microlearning promotes just-in-time learning and reinforces knowledge through repetition, this barely provides hands-on, real-world experiences. Like, in medical education, simulation-based training and patient interactions are critical for developing diagnostic and decision-making abilities. These outcomes can hardly be achieved solely through microlearning. Thus, although microlearning can sometimes supplement experiential learning, this study finds that this falls short in replacing immersive, real-life applications that reinforce theoretical knowledge across various domains.

For example, a recent case in Adelaide University in 2024 shows the tensions surrounding digital-first learning approaches. They announced plans to eliminate traditional face-to-face lectures by 2026, while opting for entirely digital learning experiences. This decision faced backlash from faculty and academic unions, including the National Tertiary Education Union, cautioning that the shift would erode academic collaboration and weaken campus culture. Many renowned professors argued that removing in-person lectures would limit meaningful student-faculty interactions and reduce the sense of academic community, thus describing the risks of over-reliance on digital microlearning formats. Therefore, this study advocates for a balanced pedagogical approach by integrating microlearning with traditional and experiential learning methods. This policy shall ensure that young adults develop both theoretical understanding and practical competencies in an engaging and interactive manner.

## 7 Conclusions

The present study investigated the need of designing customized microlearning strategies to be tailored to discipline-specific requirements of university students. Through pre- and post-intervention analyses, this study assessed the impact of microlearning on five major soft skills, namely TWS, LS, CS, TMS, and EI, across academic disciplines for university students, whereas this study addressed a research gap on whether the effectiveness of microlearning would vary across four major disciplines, namely HA, BS, MS, and TE. After formulating five hypotheses to identify which student groups showed the most significant improvements in each soft skill and how microlearning should be optimized for different academic fields, this study collected responses from 384 Chinese university students with an overall data recovery rate of 93.23%, and thus conducted major statistical analyses of current datasets.

This study found that leadership-oriented modules would be crucial for TE and MS groups of students, and emotional intelligence training is particularly valuable for students in BS group. Also, this study determined how microlearning would bridge the gap between academic training and industry expectations by linking specific soft skills to workforce demands. CS and TMS among BS and TE students aligned with corporate project management needs, whereas leadership growth in TE and MS students promoted the managerial roles. These findings provided valuable insights for educators, curriculum designers, and industry leaders, thus enabling them to refine learning methodologies, enhance employability outcomes, and strengthen academic-industry collaborations.

Some key actions that maximize the benefits of microlearning for stakeholders include the integration of structured microlearning into curricula to enhance soft skills across disciplines by university administrators. Professors should incorporate scenario-based modules into traditional teaching to boost student engagement, whereas recruitment at any higher educational institutions should prioritize candidates with microlearning experience and develop industry-specific training programs. Moreover, EdTech developers need to advance AI-driven, interactive microlearning solutions for personalized learning, whereas students can actively participate in microlearning for continuous self-improvement. This way, this study could globally establish the impact of microlearning in promoting equitable education, workforce readiness, and innovation, thus making this a vital tool for closing skill gaps and strengthening competency-based education.

### 7.1 Limitations of this study and future research scopes

Like any study on the role of microlearning, this study should acknowledge several limitations, including the following:

- This study focused on specific disciplines (namely, BS, TE, MS, and HA), which limited the applicability of current findings to other academic fields with different educational structures. Thus, future researchers can be more subject specific to yield more impactful insights.
- This study assessed the impact of microlearning on soft skills over a relatively short period, while leaving the long-term retention of these skills among young adults uncertain. In future, one should explore whether these improvements persist over time. Additionally, this study focused solely on Chinese university students, whose cultural background typically differed from other nations. To enhance generalizability, future studies should consider cross-country comparisons to examine how the effectiveness and needs of microlearning intervention shall vary across diverse educational and cultural contexts.
- A significant portion of data collection from Chinese university students relied on self-assessments, which could have introduced biases based on participants' perceptions rather than objective skill measurements. Therefore, to ensure a more accurate evaluation of the real-life impact of microlearning on young adults across disciplines, future studies should incorporate professors' assessments alongside students' self-evaluations.
- This study could barely establish a universally applicable microlearning model, while making this challenging to implement consistent interventions across different academic disciplines and institutions, the issue of which needs to be tackled in future. Besides, the present study planned to like microlearning outcomes to industry expectations, even though the direct validation from employers regarding students' improved performance in workplace settings could barely be examined.

## Data Availability

The original contributions presented in the study are included in the article/supplementary material, further inquiries can be directed to the corresponding author.
